# Multi-Element Analysis of Sicilian Citrus Fruits from Volcanic and Non-Volcanic Areas for Geographical Authentication and Food Safety Assessment

**DOI:** 10.3390/foods15173008

**Published:** 2026-08-26

**Authors:** Giuseppa Di Bella, Vincenzo Nava, Angela Giorgia Potortì, Roberto Sturniolo, Vincenzo Lo Turco

**Affiliations:** 1 Department of Biomedical and Dental Sciences and of Morphological and Functional Images (BIOMORF), University of Messina, Viale Palatucci 13, 98168 Messina, Italy; gdibella@unime.it (G.D.B.); vnava@unime.it (V.N.); roberto.sturniolo@studenti.unime.it (R.S.); vloturco@unime.it (V.L.T.); 2 Department of Chemical, Biological, Pharmaceutical, and Environmental Sciences (CHIBIOFARAM), University of Messina, Viale F. Stagno d’Alcontres 31, 98166 Messina, Italy

**Keywords:** citrus fruits, elemental fingerprint, volcanic soils, geographical characterization, food safety, chemometrics

## Abstract

This study investigated the elemental composition along the soil–plant continuum of Sicilian citrus fruits grown in volcanic (Stromboli and Etna) and non-volcanic (Marsala) areas. Following microwave-assisted acid digestion, macro- and trace elements were quantified via ICP-MS in soils, whole fruits, peel, pulp, and juice. Citrus fruits from volcanic areas generally showed higher concentrations of K, P, Fe, and Al than those from Marsala. Potentially toxic elements in edible fractions were generally low, with Pb and Cd below the applicable regulatory limits. Elemental partitioning showed higher measured concentrations of several potentially toxic elements in the peel, with lower concentrations in pulp and juice. Principal Component Analysis (PCA) revealed separation of samples according to geographical origin, while Linear Discriminant Analysis (LDA) achieved complete classification within the investigated dataset, with K, Al, Mg, and Ni. These findings indicate that elemental fingerprinting may support geographical characterization, together with nutritional and toxicological assessment.

## 1. Introduction

Citrus fruits (genus *Citrus* L., family *Rutaceae*) represent one of the most important fruit crops worldwide. With an annual production exceeding 105 million metric tons, citrus fruits represent a crucial component of the human diet and international trade [1]. Within the food sector, these fruits are consumed fresh or processed into products such as juice, pulp, purée, or frozen fruit. The essential oils extracted from them are widely used in cosmetic applications, while bioactive compounds isolated from their peel or pulp are employed in pharmaceutical applications [2,3,4].

Beyond their well-known contribution of the intake of vitamin C, phenolic compounds with antioxidant activity, and dietary fiber, citrus fruits also provide macro- and micronutrient minerals [1,2,5,6]. In particular, citrus fruits supply appreciable amounts of potassium, calcium, and magnesium, as well as trace elements such as iron, zinc, and selenium, which, although present in trace amounts, contribute to enzymatic processes, electrolyte homeostasis, and protection against oxidative stress. This mineral profile underlines their importance not only for basic nutrition but also in the prevention of chronic degenerative diseases [7,8].

Sicily is one of the main citrus-producing regions in Italy. In the 2023 campaign, Sicilian orchards yielded about 2.2 million tons of citrus fruits, accounting for approximately 70% of Italy’s total production (3.1 million tons). Italian citrus production represented approximately 30% of European production (10.7 million tons) and about 1.8% of global citrus production (169.4 million tons). Italy ranks as the 14th largest citrus exporter globally, with Sicily making a major contribution to national citrus production and export [9,10]. Given the magnitude of this production, ensuring the authenticity, geographical traceability, and safety of these fruits is important for supporting food quality control and consumer protection.

While inorganic element levels in citrus fruits are influenced by multiple factors, including cultivar genotype, agricultural management, climatic conditions, and fruit maturity at harvest [11,12,13,14,15,16,17,18,19], soil pedology and geochemistry are recognized as important factors affecting mineral uptake.

Sicily is characterized by a high degree of pedoclimatic heterogeneity, with extensive areas influenced by volcanic activity, particularly from Mount Etna and the Stromboli volcanic system in the Aeolian archipelago. The resulting volcanic soils, together with a Mediterranean climate and longstanding agronomic expertise, provide favorable conditions for high-quality citrus cultivation. Volcanic soils are characterized by high fertility and natural enrichment in trace elements, giving them distinctive physicochemical properties [20,21]. However, the physicochemical characteristics of these soils can exert a dual influence on agriculture: while providing high natural fertility through amorphous aluminosilicates, they can also alter nutrient bioavailability and promote the mobilization of trace and potentially toxic elements within the soil–plant system [21,22].

Studies of other crops grown in diverse geological settings, including volcanic areas in Sicily, have revealed significant variations in element concentrations. For instance, *Boletus aereus* and *Clitopilus prunulus* fungi collected in Sicily exhibit notable differences between volcanic and sedimentary sites [23]. Similarly, grapes cultivated on volcanic soils in Fogo Island (Cape Verde) exhibit distinct bioaccumulation patterns and enrichment factors for various elements [24].

Interest in elemental content in agricultural products, especially those grown in geologically active areas, is not limited to nutritional aspects, but also includes toxicological considerations. Indeed, the transfer of toxic and potentially toxic elements and metalloids to fruits can pose risks to human health when concentrations exceed safe thresholds [25]. According to Commission Regulation (EC) No 915/2023, only two elements in citrus fruits are subject to maximum limits: lead and cadmium. The established maximum content for lead in citrus fruits, classified as “fruit other than cranberries, currants in clusters, elderberries, and arbutus fruits”, is 0.10 mg/kg. For cadmium, citrus fruits are specifically listed, and the established maximum level is 0.020 mg/kg. In both cases, the maximum levels refer to fresh weight, after washing and separating the edible portion [26].

It is also important to note that mineral elements are not distributed uniformly within the fruit. Understanding this compartmentalized distribution and mapping element transfer from soil to distinct fruit fractions (peel, pulp, juice) are fundamental for characterizing elemental partitioning along the soil–plant continuum and evaluating differences among fruit compartments. The peel of citrus fruits, although predominantly considered nonedible, has been identified as a macro- and micronutrient-rich portion, often exceeding the concentrations found in the pulp [19,27]. This heterogeneous elemental distribution is relevant not only for estimating the nutritional contribution of the edible fractions but also for evaluating the potential valorization of citrus by-products, particularly the peel, as sources of functional compounds or mineral-rich extracts [2,20,28,29].

The characterization of elemental partitioning within the soil–plant continuum requires highly sensitive multielement analytical techniques. Inductively coupled plasma mass spectrometry (ICP-MS) has become the reference approach for food elemental analysis because of its low detection limits, wide dynamic range, and capability to determine multiple elements simultaneously. These features make ICP-MS particularly suitable for investigating elemental distribution among fruit tissues and for applications in food traceability and safety.

The multielement datasets generated via ICP-MS can be analyzed using chemometric approaches to identify geographical patterns and support food traceability. Several studies in food traceability have shown that various food products, including citrus, can be differentiated according to their geographic origin using chemical-composition data, particularly multielement fingerprints, combined with chemometric analysis [12,17,18,30,31]. More recent research has extended this approach by integrating multielement profiling with chemometric and machine-learning methods for the geographical authentication of citrus fruits [32,33,34,35]. Among these, Principal Component Analysis (PCA) and Linear Discriminant Analysis (LDA) are widely used to explore multivariate datasets, identify geographical patterns, and evaluate sample discrimination according to geographical origin [36].

Although the elemental composition of citrus fruits has been investigated previously, most studies have focused on individual plant tissues, citrus leaves, or commercially available fruits with unknown cultivation soils. Consequently, a systematic investigation of the entire soil–plant continuum, integrating soil composition with the elemental distribution in whole fruits, peel, pulp, and juice, remains largely unexplored. Addressing this gap can improve the understanding of geographical variability in citrus mineral composition while simultaneously providing information relevant to food authenticity and toxicological safety.

Therefore, the present study was designed as a proof-of-concept investigation to explore whether elemental fingerprinting combined with chemometric analysis could discriminate citrus fruits cultivated on contrasting geological substrates. Specifically, the present study aims to: (i) determine the concentrations of macro-, trace, and potentially toxic elements in cultivation soils, whole fruits, and fruit compartments (peel, pulp, and juice) of different citrus species grown in volcanic (Etna and Stromboli) and non-volcanic (Marsala) areas of Sicily; (ii) compare the elemental profiles of whole fruits from the different geographical areas using chemometric approaches; (iii) investigate elemental partitioning and transfer patterns along the soil–plant continuum by evaluating the distribution of elements from soils to whole fruits and among fruit compartments; and (iv) assess the nutritional relevance and toxicological safety of the edible fractions.

## 2. Materials and Methods

### 2.1. Chemicals and Standard Solutions

Ultrapure deionized water (10 MΩ·cm, J.T. Baker, Milan, Italy) was used throughout all procedures. Concentrated nitric acid (65% *v*/*v*, trace metal analysis grade; J.T. Baker, Milan, Italy) was used for glassware cleaning and sample digestion.

Stock standard solutions of mineral elements were purchased as follows: Al, As, Cr, Cu, Fe, Ni, Pb, Se, and Zn (1000 mg·L^−1^ in 2% nitric acid) from Fluka (Milan, Italy), and Ca, Cd, Co, Hg, K, Mg, Mn, Mo, Na, P, Ti, and V (1000 mg·L^−1^ in 2% nitric acid) from Merck (Darmstadt, Germany). Calibration standard solutions were prepared in matrix-specific concentration ranges for citrus and soil samples.

Internal standardization was performed using a multielement standard solution containing ^45^Sc, ^73^Ge, ^115^In, and ^209^Bi (1000 mg·L^−1^ in 2% HNO_3_; Fluka, Milan, Italy), added on-line at a concentration of 1.5 mg·L^−1^ to correct for instrumental drift and matrix effects. A Re stock solution (1000 mg·L^−1^ in 2% HNO_3_; Fluka, Milan, Italy) was used as a preparation standard at 0.8 mg·L^−1^ to assess digestion efficiency and correct for any volumetric variations. Instrument tuning was performed using an ICP-MS tuning solution (Agilent, Santa Clara, CA, USA) containing 1 µg·L^−1^ of ^7^Li, ^59^Co, ^89^Y, and ^205^Tl in 2% HNO_3_.

High-purity argon (N 5.0, 99.9990%) and helium (N 5.5, 99.9995%) were supplied by Rivoira Gases (Milan, Italy) and used as plasma and collision/reaction gases, respectively.

For method validation, certified reference materials appropriate for the investigated matrices were employed: SRM 1570a (spinach leaves) for citrus samples, obtained from the National Institute of Standards and Technology (NIST, Gaithersburg, MD, USA), and CRM048 (sandy soil) for soil samples, obtained from Sigma-Aldrich RTC, Inc. Laramie, WY, USA).

### 2.2. Instrumentation

Triplicate sample digestions were performed using a closed-vessel microwave system (Ethos 1; Milestone, Bergamo, Italy), equipped with PTFE vessels (maximum pressure 110 bar) and operating at a maximum microwave power of 1600 W under temperature- and pressure-controlled conditions.

Multielement determination was performed using an iCAP-Q ICP-MS system (Thermo Fisher Scientific, Bremen, Germany), equipped with a classic Fassel-type torch (2.5 mm i.d.), a ShieldTorch system, a PFA cyclonic spray chamber, and a concentric nebulizer (6 mm o.d.) (all from Thermo Fisher Scientific, Bremen, Germany), as well as an ASX-520 autosampler (Cetac Technologies Inc., Omaha, NE, USA). Nickel sampler and skimmer cones (1.1 mm and 0.5 mm, respectively) were employed. The QCell collision/reaction cell was operated either in no-gas mode or in kinetic energy discrimination (KED) mode using helium as the collision gas. Mn, Zn, Hg, Cd, and Pb were determined in no-gas mode, whereas the other elements were acquired in He-KED mode to minimize spectral interferences. The monitored isotopes were ^23^Na, ^24^Mg, ^27^Al, ^31^P, ^39^K, ^40^Ca, ^48^Ti, ^51^V, ^52^Cr, ^55^Mn, ^56^Fe, ^59^Co, ^60^Ni, ^63^Cu, ^66^Zn, ^75^As, ^78^Se, ^98^Mo, ^114^Cd, ^121^Sb, ^202^Hg, and ^208^Pb.

### 2.3. Study Sites and Environmental Characteristics

**Figure 1:**
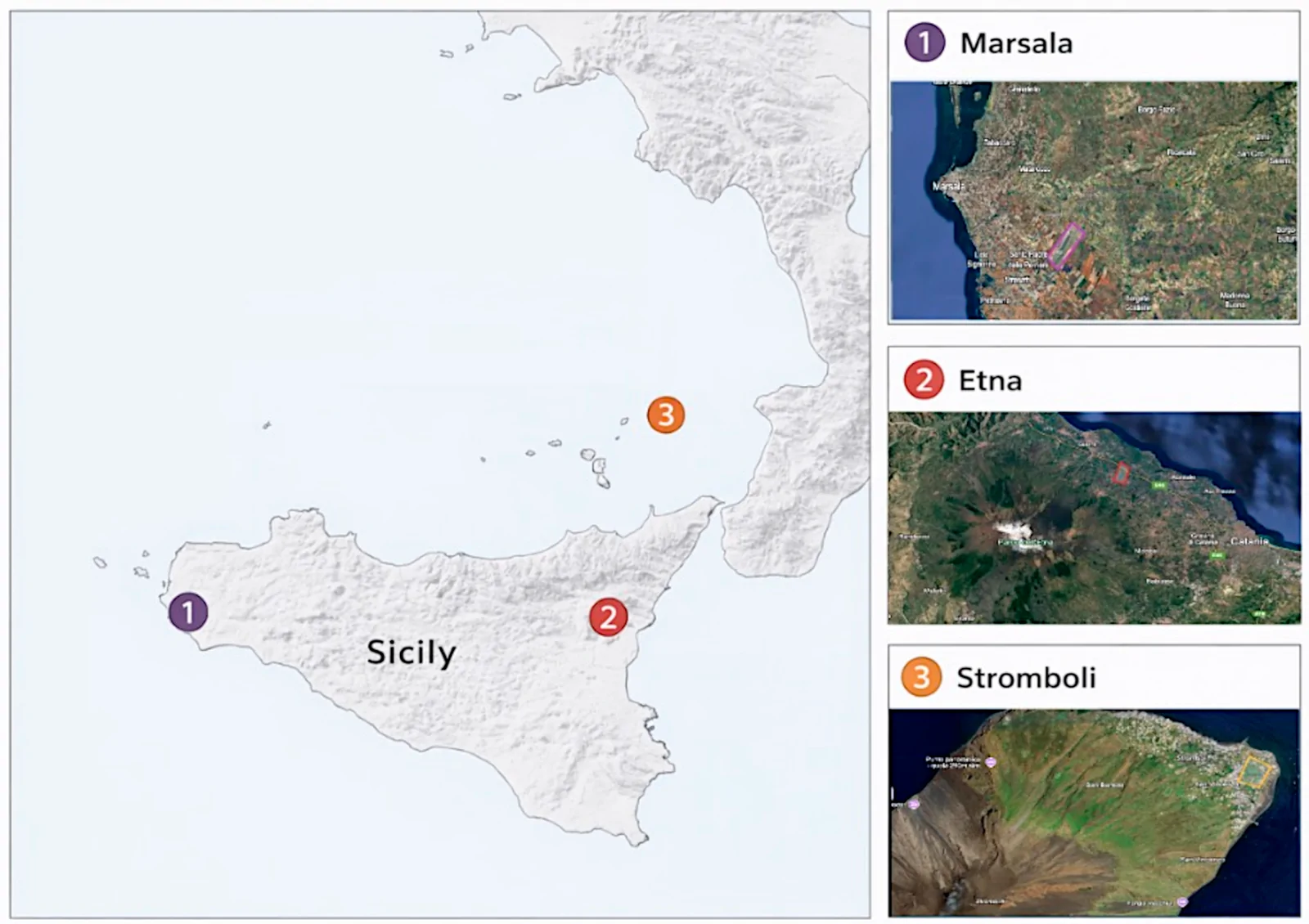
Geographical location of the three citrus orchards and corresponding soil sampling sites in Sicily (Marsala, Mount Etna, and Stromboli).

The study was carried out on citrus fruits and their cultivation soils collected from both volcanic and non-volcanic areas in Sicily (southern Italy). The volcanic areas included two distinct sites: one located on the island of Stromboli, part of the Aeolian Archipelago in the province of Messina, and the other on the flanks of Mount Etna, on the eastern coast of Sicily in the province of Catania. The non-volcanic site was located in Marsala, a coastal area in the province of Trapani (western Sicily) (Figure 1). These three sites were selected to represent contrasting geological environments while remaining within the same regional context of Sicily.

From a geological standpoint, the Stromboli soils derive from recent basaltic pyroclastic deposits (fine-grained ash, lapilli, and scoria) overlying older lava flows. This stratigraphy results in a highly porous and well-drained volcanic topsoil [37]. On Mount Etna, soils have formed on successive layers of lava flows and tephra deposits. This stratified volcanic substrate results in a heterogeneous soil matrix composed of coarse gravel, sand, and silty-clay fractions, with fertility levels varying according to the local depositional history and degree of weathering [38]. In contrast, the soils of Marsala have formed on marine Pleistocene and Holocene deposits (calcarenite, organogenic sands, and clay-rich marls), resulting in a denser and calcareous topsoil with moderate drainage capacity [39].

The three sampling sites were geographically delineated as follows: the Stromboli citrus orchard is situated at 38°48′10″ N, 15°14′25″ E, at an elevation of 2–5 m above sea level (a.s.l.); the Mount Etna orchard lies at 37°40′34″ N, 15°08′51″ E, on the volcano’s flank between 200 and 300 m a.s.l.; and the Marsala orchard is positioned at 37°44′22″ N, 12°33′07″ E, on coastal sediments rising from 50 to 150 m a.s.l. The investigated cultivation areas covered approximately 3.06 ha in Stromboli, 7.01 ha on Mount Etna, and 4.96 ha in Marsala.

During the fruit development and harvest period (July–December 2024), cumulative rainfall was 558 mm at Stromboli, 1055 mm on Mount Etna, and 324 mm at Marsala [40]. All three sites are characterized by Mediterranean climatic conditions, although they differ in altitude and rainfall regime. The reported geological and climatic characteristics provide the environmental context of the investigated sites and should be considered when interpreting differences in elemental composition among geographical areas.

The three orchards were selected to represent contrasting geological settings. All sites consisted of non-commercial orchards managed according to traditional local practices and included the same citrus species investigated in the present study, thereby reducing variability related to species composition.

### 2.4. Samples

A total of 12 soil samples were collected, comprising four composite soil samples from each of the three geographical areas (Stromboli, Etna, and Marsala). Each geographical area was represented by a non-commercial citrus orchard in which lemon (*Citrus limon*), mandarin (*Citrus reticulata*), yellow grapefruit (*Citrus paradisi*, yellow variety), and pink grapefruit (*Citrus paradisi*, pink variety) were grown under traditional local management practices. Soil samples were collected in accordance with the Official Methods of Soil Chemical Analysis (Italian Ministerial Decree, 13 September 1999). The four composite soil samples collected from each cultivation site were used to characterize the local pedological background. Soil samples were collected from the topsoil layer (5–30 cm depth), corresponding to the agronomically active horizon for citrus cultivation. Each composite sample was obtained by homogenizing approximately 5 kg of soil collected from multiple sampling points distributed throughout the cultivation site and including areas in which the different citrus species were grown. The collected soil was manually mixed to ensure homogeneity and subsequently reduced by quartering to obtain a representative laboratory sample of approximately 100 g.

Fruit sampling was carried out in parallel at the three cultivation sites, within the same orchards from which the corresponding soil samples had been collected. Each composite fruit sample consisted of approximately 25 kg of fruits harvested at full ripeness (November–December 2024). Fruits were collected from multiple trees distributed throughout the cultivation site to account for within-site spatial variability. For each selected tree, fruits were randomly collected from different parts of the canopy to account for within-tree variability. After thorough mixing, the bulk fruit sample was reduced by systematic subsampling to obtain a representative 2 kg analytical sample. Overall, fruit sampling yielded 39 composite samples: 12 lemons, 9 mandarins, 9 yellow grapefruits, and 9 pink grapefruits, equally distributed among the three geographical areas.

After thorough washing with deionized water and air drying, the representative analytical sample was allocated to whole-fruit analysis and to anatomical fractionation. For whole-fruit analysis, the entire fruits were cut into small pieces using a stainless-steel knife and subsequently homogenized in an agate mortar until a uniform consistency was obtained. The remaining fruits were manually separated into peel and edible tissues using a stainless-steel knife. The peel was cut into small pieces and homogenized in an agate mortar until a uniform consistency was obtained. The edible portion was divided into two fractions. One fraction was mechanically squeezed using a laboratory stainless-steel juicer to obtain juice, which was filtered through ashless filter paper to remove suspended solids and thoroughly mixed to ensure homogeneity prior to digestion. The second fraction was cut into small pieces and homogenized in an agate mortar until a uniform pulp consistency was obtained, without prior juice extraction. Overall, the sampling and fractionation procedure generated 156 analytical citrus samples, corresponding to 39 fruit samples each analyzed as four matrices (whole fruit, peel, pulp, and juice).

### 2.5. Sample Preparation

Approximately 0.5 g of each sample was weighed into an acid-washed PTFE vessel, and 1 mL of the Re preparation standard solution was added. Soil samples were digested using 2 mL of 65% HNO_3_, 4 mL of 37% HCl, and 2 mL of 48% HF. Citrus samples were digested using 8 mL of 65% HNO_3_ and 2 mL of 30% H_2_O_2_. The microwave digestion program for soil samples consisted of a temperature ramp to 200 °C in 10 min, followed by a 15 min holding step. The microwave digestion program for citrus samples consisted of a temperature ramp to 180 °C in 15 min, followed by a 15 min holding step. Microwave power was automatically modulated by the instrument up to a maximum of 1000 W. Certified reference materials were digested under the same conditions.

After cooling, each sample was quantitatively transferred into a pre-cleaned 25 mL volumetric flask, brought to volume with deionized water, and stored at 4 °C. Soil samples were subsequently diluted 1:100 prior to analysis to ensure compatibility with the instrumental detection range. All sample preparations were carried out in triplicate.

### 2.6. ICP-MS Analysis

ICP-MS operating conditions are summarized below and were consistent with those previously adopted for multielement analysis [41,42,43]. The instrument was operated at an RF power of 1550 W, with a sample depth of 5 mm and an introduction flow rate of 0.93 mL/min. Argon gas flow rates were set at 14 L/min for plasma, 0.8 L/min for auxiliary, and 1.1 L/min for carrier gas. Helium was used as the collision gas at 4.7 mL/min. Additional instrumental settings included a dwell time of 1 s, extract lens 1 voltage of 1.5 V, spray chamber temperature of 2.7 °C, and a nebulizer pump speed of 0.1 rps. Integration times were set at 0.5 s per point for Fe, Se, and As, and 0.1 s per point for all other elements. Each mass was integrated over three points, with three replicate acquisitions per measurement. Samples were analyzed in analytical batches including procedural blanks and certified reference materials for quality control. Each digest was measured in triplicate.

### 2.7. Analytical Performances

Method validation was carried out in accordance with EURACHEM guidelines [44]. The analytical performance of the method was assessed by evaluating linearity, limits of detection (LOD), limits of quantification (LOQ), accuracy, repeatability, and intermediate precision. LOD and LOQ were calculated as 3.3σ/S and 10σ/S, respectively, where σ is the standard deviation of the blank signal and S is the slope of the calibration curve. Accuracy was evaluated using certified reference materials representative of the investigated matrices and, where certified values were unavailable, by spike recovery. Repeatability and intermediate precision were assessed through replicate analyses. The analytical performance data, including element-specific LOD and LOQ values, are summarized in Tables S1 and S2 of the Supplementary Materials.

### 2.8. Statistical Analysis

Statistical analyses were performed using SPSS software (version 13.0 for Windows; SPSS Inc., Chicago, IL, USA).

Three multivariate data matrices were used. The first matrix, corresponding to soil samples, had dimensions 12 × 22, representing 12 soil samples and the concentrations of 22 mineral elements. The second matrix, related to whole citrus fruits, had dimensions 39 × 22, with 39 fruit samples and the same 22 mineral elements. The third matrix, consisting of fruit fractions (peels, pulps, and juices), had dimensions 117 × 22, again with the same 22 variables measured across all samples. Whenever element concentrations were below the limit of quantification (LOQ), a value equal to half the limit of detection (LOD/2) was assigned for statistical analysis.

All concentration values were subjected to logarithmic transformation to reduce the skewness of the variable distributions and to mitigate the influence of high-magnitude outliers. The non-parametric Kruskal–Wallis test was used to assess the statistical significance of differences among geographical origins (Stromboli, Etna, and Marsala) for soils, whole fruits, and fruit fractions (peel, pulp, and juice), with citrus samples analyzed separately by species. Differences were considered statistically significant at *p* < 0.05.

Although the data were log-transformed, they were standardized prior to Principal Component Analysis (PCA) to ensure comparability across variables [45]. The suitability of the data for PCA was assessed using the Kaiser–Meyer–Olkin (KMO) measure of sampling adequacy and Bartlett’s test of sphericity. Linear Discriminant Analysis (LDA) was performed using the full set of elemental variables to identify the linear combinations that maximized the separation among the predefined geographical groups. Internal model performance was evaluated using leave-one-out cross-validation (LOOCV). Classification accuracy was evaluated for both the original and cross-validated models. Considering the relatively limited sample size, the chemometric analyses should be regarded as exploratory and intended to evaluate the discriminatory potential of elemental fingerprints within the investigated dataset rather than to develop a generally applicable predictive classification model.

### 2.9. Uptake of Mineral Elements Through Juices

The estimated daily intake (EDI) of mineral elements from citrus juice consumption was calculated by multiplying the mean sample concentration (mg·L^−1^) by a standard serving size of 200 mL (equivalent to one glass). The resulting daily intakes (mg·day^−1^) for essential elements were then compared with the applicable dietary reference values established by the EFSA [46] and EU legislation [47]. Estimated dietary exposures to potentially toxic elements were calculated for a 70 kg adult and compared with toxicological reference values (TRVs) established by the European Food Safety Authority [48,49,50,51], the Environmental Protection Agency [52], the Joint FAO/WHO Expert Committee on Food Additives (JECFA), the Agency for Toxic Substances and Disease Registry [53], and the World Health Organization [54].

## 3. Results and Discussion

### 3.1. Geographical Variation in Soil Elemental Composition

Significant differences in soil elemental composition were observed among the three cultivation sites (Table 1). The two volcanic sites (Stromboli and Mount Etna) exhibited elemental profiles that differed from those of the non-volcanic site (Marsala). Specifically, soils from Stromboli and Etna showed significantly higher concentrations of K, P, Fe, and Al than Marsala soil. For example, K concentrations were 19,044 ± 546 mg·kg^−1^ in Stromboli and 13,971 ± 1257 mg·kg^−1^ in Etna, versus 6241 ± 183 mg·kg^−1^ in Marsala. Similarly, P was 2238 ± 20 mg·kg^−1^ in Stromboli and 2500 ± 41 mg·kg^−1^ in Etna, compared to 882 ± 94 mg·kg^−1^ in Marsala. Fe and Al also followed this trend. The enrichment in K, Fe, and Al observed in the volcanic soils is consistent with previous studies describing the geochemical characteristics of volcanic soils in different geographical regions, including the Ecuadorian Andes [22]. These findings consistent with geochemical features commonly associated with volcanic environments.

Conversely, soils from Marsala showed higher concentrations of Ca, Mg, Na, Cr, and Pb than the volcanic soils. Ca levels in Marsala soil were 8705 ± 120 mg·kg^−1^, higher than 6955 ± 956 mg·kg^−1^ for Stromboli and 6734 ± 272 mg·kg^−1^ for Etna. Soils from Marsala showed the highest sodium content at 5742 ± 138 mg·kg^−1^, followed by Etna soils at 4483 ± 175 mg·kg^−1^, and Stromboli soils at 3325 ± 47 mg·kg^−1^. Cr was 48.95 ± 5.55 mg·kg^−1^ in Marsala, significantly higher than 2.68 ± 0.95 mg·kg^−1^ in Stromboli and 13.30 ± 1.32 mg·kg^−1^ in Etna. Pb concentrations were also significantly higher in Marsala soils (75.35 ± 7.87 mg·kg^−1^) than in Etna (41.40 ± 3.06 mg·kg^−1^) and Stromboli (11.65 ± 0.51 mg·kg^−1^). Etna soils showed significantly higher concentrations of Se, Cd, and Mo. Specifically, Se concentrations in Etna were approximately 9 to 15 times higher than those in Stromboli and Marsala, while Cd and Mo were at least twofold higher. This enrichment is consistent with the distinctive geochemical composition of Etna volcanic materials and the contribution of relatively recent volcanic deposits. Active volcanic emissions, including passive degassing and ash deposition, have been reported to contribute to the enrichment of surrounding soils with volatile and chalcophile elements, including Se, Cd, and Mo [55,56].

In contrast, Stromboli soils showed significantly higher concentrations of K and Al than Etna and Marsala. Although Stromboli Zn content exceeds that of Marsala, it is lower than that of Etna, showing a different site-specific pattern for this element.

These differences in soil elemental composition provide a relevant context for interpreting the elemental profiles observed in the corresponding citrus fruits.

### 3.2. Geographical Variation in Citrus Fruits Elemental Composition

The results for whole fruits are presented in Table 1 alongside those for the corresponding cultivation soils.

Consistent with the differences observed in the corresponding cultivation soils, citrus fruits grown in the volcanic areas (Stromboli and Etna) generally exhibited elemental profiles that differed from those observed in the non-volcanic area (Marsala). These differences are consistent with the contrasting geological characteristics of the investigated cultivation sites, while site-specific environmental and agronomic factors may also have contributed to the observed variability.

Citrus fruits from volcanic areas generally exhibited significantly higher concentrations of K and P when compared to fruits from Marsala. This trend is consistent with the higher concentrations of K and P measured in the corresponding volcanic soils. Specifically, this trend was observed across different citrus types. For instance, lemons from Stromboli contained 2026 ± 10 mg·kg^−1^ of K and 242 ± 6 mg·kg^−1^ of P, while those from Etna contained 1871 ± 12 mg·kg^−1^ of K and 234 ± 3 mg·kg^−1^ of P. In contrast, lemons from Marsala showed significantly lower levels of K (1642 ± 13 mg·kg^−1^) and P (173 ± 5 mg·kg^−1^). Similarly, yellow and pink grapefruits from Stromboli and Etna showed higher K and P concentrations than those from Marsala. The trend of higher P in volcanic regions was also observed in Mandarin, whereas a slightly different pattern was observed for K, with the Marsala value comparable to that of Etna, though still lower than that of Stromboli. Relatively high P concentrations were measured in citrus fruits grown on the investigated volcanic soils, despite the well-known phosphorus-fixing capacity of volcanic Andisols [22].

Conversely, Ca concentrations generally present an inverse trend. Citrus fruits cultivated in volcanic areas consistently exhibit lower Ca concentrations compared to those grown in Marsala, which is consistent with the lower Ca concentrations measured in the corresponding soils. For example, mandarin fruits from Stromboli and Etna contained 282 ± 2 mg·kg^−1^ and 236 ± 2 mg·kg^−1^ of Ca, respectively, while Marsala mandarins contained 374 ± 1 mg·kg^−1^. An exception to this trend was observed in pink grapefruits, where Marsala fruits contained lower Ca (217 ± 1 mg·kg^−1^) than those from Stromboli (290 ± 4 mg·kg^−1^) and Etna (320 ± 4 mg·kg^−1^). This deviation highlights that soil mineral abundance is not the sole determinant of fruit composition. As documented in the literature, elemental accumulation in citrus is known to be cultivar-dependent and may be influenced by genetic factors, rootstock interactions, and selective physiological uptake [1,2]. The higher Ca concentrations observed in pink grapefruit, despite the lower Ca content of the corresponding soils, further suggest that factors other than total soil Ca concentration may contribute to the elemental composition of the fruit.

Lemons and mandarins from the volcanic area of Stromboli showed higher Na concentrations than those from Etna and Marsala. For yellow and pink grapefruits, higher Na concentrations were observed at both volcanic sites.

This pattern contrasts with the elemental composition of the soils in these regions. Soils from Marsala showed the highest Na content, followed by soils from Etna and Stromboli. This contrasting distribution of Na between soils and fruits suggests that fruit Na concentrations cannot be explained solely by total soil Na concentrations and may also depend on factors affecting element availability and plant uptake.

Pink grapefruits from volcanic areas showed higher Fe levels. Concentrations ranged from 3.29 ± 0.09 mg·kg^−1^ (Stromboli) to 3.75 ± 0.07 mg·kg^−1^ (Etna), notably surpassing 1.99 ± 0.03 mg·kg^−1^ found in Marsala. Similarly, Mn concentrations were elevated in fruits from volcanic regions, with Etna showing the highest levels at 0.60 ± 0.04 mg·kg^−1^, followed by Stromboli at 0.51 ± 0.02 mg·kg^−1^. Both volcanic sites showed significantly higher Mn concentrations than Marsala, where the concentration was 0.27 ± 0.01 mg·kg^−1^. The only significant difference observed for Zn was in yellow grapefruit. The Zn content of Etna yellow grapefruit was higher than those from Stromboli and Marsala.

Many trace elements, including Cu, Al, Mo, Se, Ni, V, Co, and Hg, were generally present at higher concentrations in citrus from Stromboli and Etna than in those from Marsala, where they were often very low or below the LOQ.

Despite significantly higher concentrations of Cr, As, and Pb in Marsala soils than in those in volcanic soils, their levels in Marsala fruit parts were generally very low or at the limit of quantification. This discrepancy suggests that total soil concentrations alone do not determine the levels of these elements in citrus fruits. As reported in the literature, soil physicochemical properties can strongly influence trace-element mobility and bioavailability, thereby affecting their transfer to plants [21,22]. However, these parameters were not specifically investigated in the present study, and the mechanisms underlying the observed differences cannot be conclusively established.

Despite Cd being present in Marsala soil, levels of Cd in Marsala citrus fruits are consistently below the quantification limit, whereas higher concentrations were observed in fruits from the volcanic sites, particularly Etna. These findings further show that the relationship between soil and fruit Cd concentrations varied among the investigated sites and cannot be interpreted solely on the basis of total soil concentration.

Taken together, the results highlight distinct patterns of elemental composition across citrus species and cultivation sites. Lemon consistently exhibited higher K and Fe concentrations across the investigated cultivation sites. Yellow grapefruit generally exhibited higher Ca concentrations, particularly in Marsala, whereas samples from Etna showed comparatively higher Zn and Se concentrations. Pink grapefruit frequently showed elevated levels of several macronutrients and micronutrients, particularly at Etna and Stromboli. Across all varieties and cultivation sites, potentially toxic elements were generally present at low concentrations or below LOQ.

Despite the marked difference in cumulative rainfall during the fruit development and harvest period between the two volcanic cultivation sites (approximately 558 mm at Stromboli and 1055 mm at Etna), both sites exhibited broadly similar elemental patterns and clustered together in the multivariate analyses, whereas the non-volcanic site (Marsala) remained clearly separated. Although citrus species and harvesting period were standardized across the three geographical areas, other environmental variables, including local climatic conditions and agronomic practices, were not independently controlled. Therefore, the individual contribution of geological substrate cannot be completely disentangled from other site-specific factors. Nevertheless, the observed similarities between the two volcanic sites despite their contrasting rainfall regimes are consistent with the hypothesis that geological background may contribute to the elemental profiles of the investigated citrus fruits.

### 3.3. Elemental Distribution Within Citrus Fruit

The results concerning the distribution of elements within the fruit are presented in Table 2. Overall, differences among geographical origins were more evident than those among citrus species in the elemental composition of peel, pulp, and juice than in botanical variety. On an as-is basis, most elements showed a recurrent concentration gradient across fruit fractions, with higher measured concentrations in the peel, intermediate concentrations in the pulp, and lower concentrations in the juice. However, because moisture content was not determined, these differences cannot be interpreted as evidence of preferential elemental accumulation or tissue-specific partitioning on a dry-matter basis. Accordingly, the observed pattern should be considered descriptive of the elemental concentrations measured in the analyzed fruit fractions. For the edible fractions, these as-is concentrations remain directly relevant to nutritional and toxicological assessments.

Compared to the peels of other citrus fruits, lemon peels are characterized by higher concentrations of K and Fe, especially in samples from Stromboli and Etna. Yellow and pink grapefruit peels from Etna and Stromboli showed higher concentrations of Al and Mo. Etna yellow grapefruit peels also showed higher concentrations of Zn and Se, while pink grapefruit peels from the same site showed higher concentrations of Ni, Cr, Pb, and Hg.

V, Co, Pb, As, Sb, Ti, Cd, and Hg were below the LOQ in almost all the juices analyzed. Exceptions were Co in yellow and pink grapefruit juices from fruits cultivated on volcanic soils and Cd in pink grapefruit from Etna. The pink and yellow grapefruits from both volcanic sites showed higher concentrations of Se, Zn, and Al.

Table S3 of the Supplementary Materials reports the transfer rates of individual elements from soil to peel, pulp, and juice for each geographical area, calculated using the measured concentrations without moisture correction of the fruit fractions. Among the investigated elements, K and P exhibited the highest transfer rates. K and P also showed consistently higher transfer rates across all fruit fractions in samples from Marsala relative to those from Stromboli and Etna. A similar trend was observed for Ca, Zn, and Cu across all fruit fractions.

Some elements exhibited different transfer patterns between volcanic and non-volcanic sites. For example, the transfer rates of Mo were generally higher in citrus fruits from Stromboli and Etna than in those from Marsala, particularly in yellow and pink grapefruit. Hg showed measurable transfer rates in the peel and pulp of citrus fruits from the volcanic sites, whereas in lemon measurable transfer was observed only in the peel. Similarly, but with much lower values, Co, Pb, As, Sb, Ti, and Cd showed higher transfer rates in samples from the volcanic sites, especially in peel and pulp. Additionally, generally low transfer rates were observed for V, Fe, Al, and Mn.

Overall, the low transfer rates observed for several trace elements were consistent with previous studies on citrus fruits reporting limited soil-to-fruit transfer for a number of metals, although direct quantitative comparisons are constrained by differences in species, soil characteristics, and calculation basis [57,58].

To evaluate compliance with current food safety regulations, Pb and Cd concentrations measured in the edible fractions were compared with the maximum levels established by Commission Regulation (EU) 2023/915.

For Pb, the maximum permissible level in citrus fruits was set at 0.10 mg·kg^−1^. Across all citrus species and cultivation sites examined, the concentrations of Pb in the edible parts (pulp and juice) were consistently well below this regulatory limit. For instance, pink grapefruit pulp from Etna contained 0.017 ± 0.006 mg·kg^−1^ of Pb, while its juice from the same location showed Pb concentrations below the LOQ.

Regarding Cd, the maximum permissible level in citrus fruits is 0.020 mg·kg^−1^. The Cd concentrations measured in the edible parts of the citrus fruits were below this specified limit. The highest value was determined for the pulp of Etna lemons, with a mean concentration of 0.015 ± 0.004 mg·kg^−1^.

### 3.4. PCA-Based Geographical Discrimination of Soil and Citrus Samples

PCA was applied to explore the multivariate structure of the soil and whole-fruit datasets. The analysis was performed on both soil samples (matrix: 12 samples × 11 variables) and whole citrus fruit samples (matrix: 39 samples × 22 variables). Before PCA, a preliminary variable selection step was applied to the soil dataset to obtain a positive definite correlation matrix and ensure suitability for factor extraction. Variables were screened using the non-parametric Kruskal–Wallis test according to their statistical significance (*p* < 0.05) in the comparison among the three geographical areas, and those associated with the lowest *p*-values were retained. The final variable set included K, P, Mg, Na, Fe, Cu, Mn, Al, Cr, Pb, and As. This preliminary screening inherently reflects the geographical differences of the selected variables. Therefore, the subsequent PCA on the soil dataset was not employed as an unsupervised exploratory method to independently prove geographical discrimination. Rather, it was used as a multivariate projection tool to visually illustrate the spatial relationships and intra-group variance associated with these specific geochemical markers. PCA was then performed on this reduced variable set. The suitability of the data for factor analysis was subsequently assessed separately for the soil and fruit datasets. The Kaiser–Meyer–Olkin (KMO) measure of sampling adequacy was 0.741 for the soil dataset and 0.748 for the citrus dataset, both exceeding 0.7, which is generally interpreted as indicative of good sampling adequacy. Additionally, Bartlett’s test of sphericity indicated a chi-square value of 256.992 for the soil samples and 1895.213 for the fruit samples, with *p* < 0.001 in both cases. These results suggest that the correlation matrices were suitable for PCA.

The following discussion pertains to the PCA performed on the soil dataset. According to the Kaiser criterion, two principal components with eigenvalues greater than one (9.119 and 1.543) were extracted. They explained 82.903% and 14.029% of the total variance, respectively, accounting for a cumulative variance of 96.932%. The factor loadings for the two extracted components and the communalities for each original variable are reported in Table S4 of the Supplementary Materials. The extracted components adequately represented all variables, since no variables with low factor loadings were identified and the communalities were consistently ≥0.883. These high communalities reflect the strong inter-variable correlations within the investigated soil dataset.

**Figure 2:**
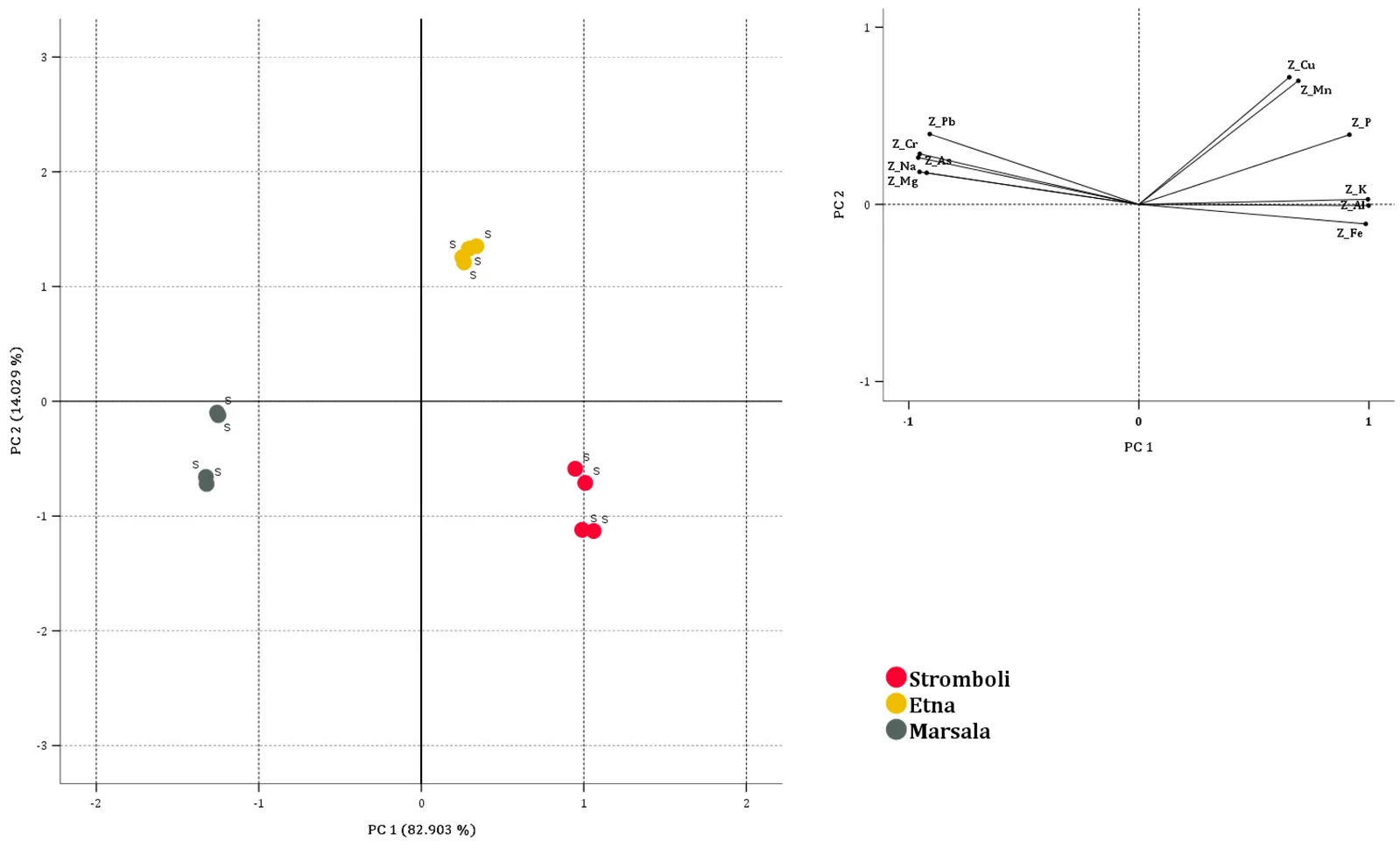
PCA score plot of soil samples (*n* = 12) by origin (Stromboli, Etna, Marsala) with loading vectors.

Figure 2 illustrates the two-dimensional scatter plot of the samples, along with the corresponding factor loadings. Al, K, Fe, and P showed a strong positive correlation with PC1, whereas Na, Mg, Cr, As, and Pb are negatively correlated with PC1. On the other hand, Cu and Mn showed a positive correlation with PC2. The score plot showed a clear separation of soil samples from Stromboli, Etna, and Marsala. Soils from Etna, located in the positive quadrant of both PC1 and PC2, were associated with higher concentrations of Cu and Mn, while soils from Stromboli, in the positive PC1 and negative PC2 quadrant, were associated with higher concentrations of Al and Fe.

For whole citrus fruit samples, PCA extracted five principal components with eigenvalues greater than one, according to the Kaiser criterion. These components collectively explained 93.326% of the total variance, with the first component (PC1) accounting for 60.559%, and the others contributing as follows: PC2 for 11.981%, PC3 for 9.458%, PC4 for 6.687%, and PC5 for 4.641%. The detailed component loadings and the communalities (*h*^2^) for each original variable are provided in Table S5 of the Supplementary Materials. The results indicated that all variables were adequately represented in the reduced component space, as suggested by the high communalities (≥0.836) and the absence of variables with weak factor loadings.

**Figure 3:**
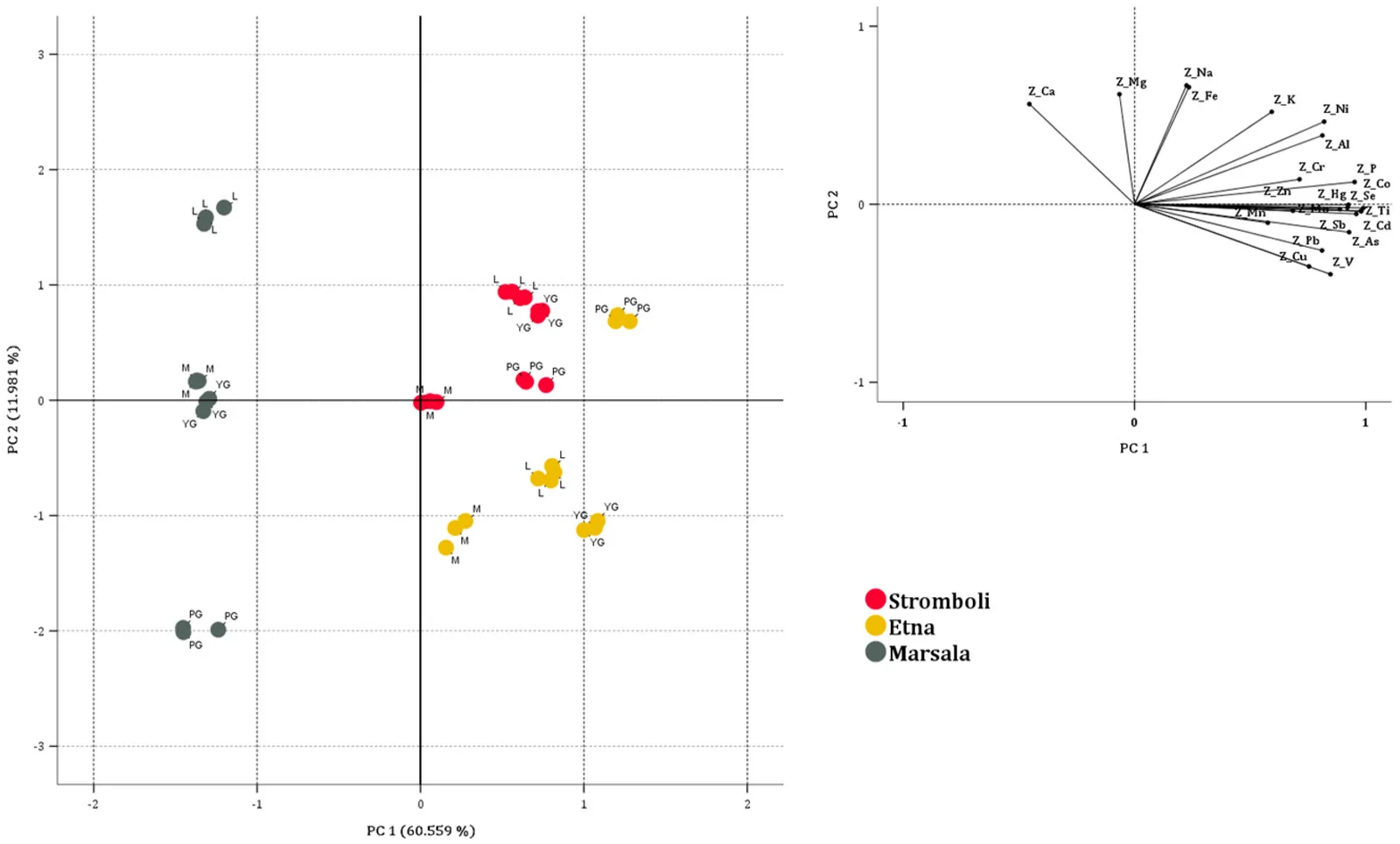
PCA score plot of citrus samples (*n* = 39) by origin (Stromboli, Etna, Marsala) with loading vectors.

The biplot in Figure 3 displays the spatial distribution of whole citrus samples along the two principal components, as well as the directional vectors corresponding to each variable.

PC1 showed strong positive associations with Co, Ti, Cd, Sb, Pb, As, Hg, Se, Mo, V, Ni, Al, Cu, Cr, Zn, K, and Mn, while Ca was negatively associated. PC2, on the other hand, showed mainly positive associations with Na, Fe, Mg and Ca.

Geographical clustering was evident: samples from Stromboli and Etna, characterized by positive PC1, were associated with positive loadings for most elements, whereas Ca and Mg showed different loading patterns. In contrast, Marsala samples were located in a different region of the plot with negative PC1. Moreover, the samples from Stromboli and Etna showed partial separation. All Stromboli samples were in the first quadrant, while all Etna samples, except yellow grapefruit, were located in the fourth quadrant. Groupings based on citrus species (lemon, mandarin, yellow and pink grapefruit) were also evident in the PCA plot. In Marsala, differences among citrus species were more pronounced, suggesting that botanical differences may contribute to the observed elemental variability at this site.

The analysis revealed that the compositional differences between samples from volcanic and non-volcanic sites were clearly detectable in the multivariate space. Overall, PCA highlighted a clear multivariate separation between samples from volcanic and non-volcanic cultivation areas within the investigated dataset, whereas the two volcanic sites showed partially distinct elemental patterns.

### 3.5. Linear Discriminant Analysis

Following PCA, Linear Discriminant Analysis (LDA) was applied as a supervised classification technique to identify the linear combinations of variables that best discriminate the predefined geographical groups.

LDA was performed using the full set of independent variables to evaluate the global discriminant structure and the relative contribution of each parameter to group separation. This approach was preferred over stepwise selection to avoid variable exclusion based on purely statistical criteria.

LDA resulted in two canonical discriminant functions. These functions represent linear combinations of the original variables that maximize the separation among the predefined groups. The first canonical function (Function 1) accounted for 86.5% of the total discriminating ability, whereas the second function (Function 2) explained 13.5%. The corresponding eigenvalues were 423.517 and 65.824, respectively, indicating that Function 1 contributed more substantially to group discrimination. The Wilks’ lambda for the combined test of Functions 1 through 2 was 0.001 (χ^2^ = 261.452, *p* < 0.001), indicating statistically significant differences among the predefined groups. When Function 2 was tested separately, controlling for the effect of Function 1, Wilks’ lambda was 0.015 (χ^2^ = 107.153, *p* < 0.001), suggesting that both functions significantly contributed to the discrimination.

The standardized canonical coefficients showed that the most influential variables in Function 1 were K (+5.035), Al (+4.603), Mg (−4.492), Ni (−3.913), Cd (−3.578), Na (−3.464), Ca (+3.376), and V (+3.330), while Function 2 was mainly driven by Na (−8.826), K (+8.624), Ca (+7.826), Cu (+6.817), and Mg (−6.255). The full list of standardized canonical coefficients, along with the unstandardized coefficients used to compute the canonical variable scores for each case, is provided in Table S6 of the Supplementary Materials. The group centroids for Function 1 were −27.728 (Marsala), +16.989 (Etna), and +10.740 (Stromboli), indicating separation among the investigated geographical groups primarily along this axis. Function 2 centroids (+8.756 for Etna and −10.178 for Stromboli) further distinguished these two volcanic regions.

**Figure 4:**
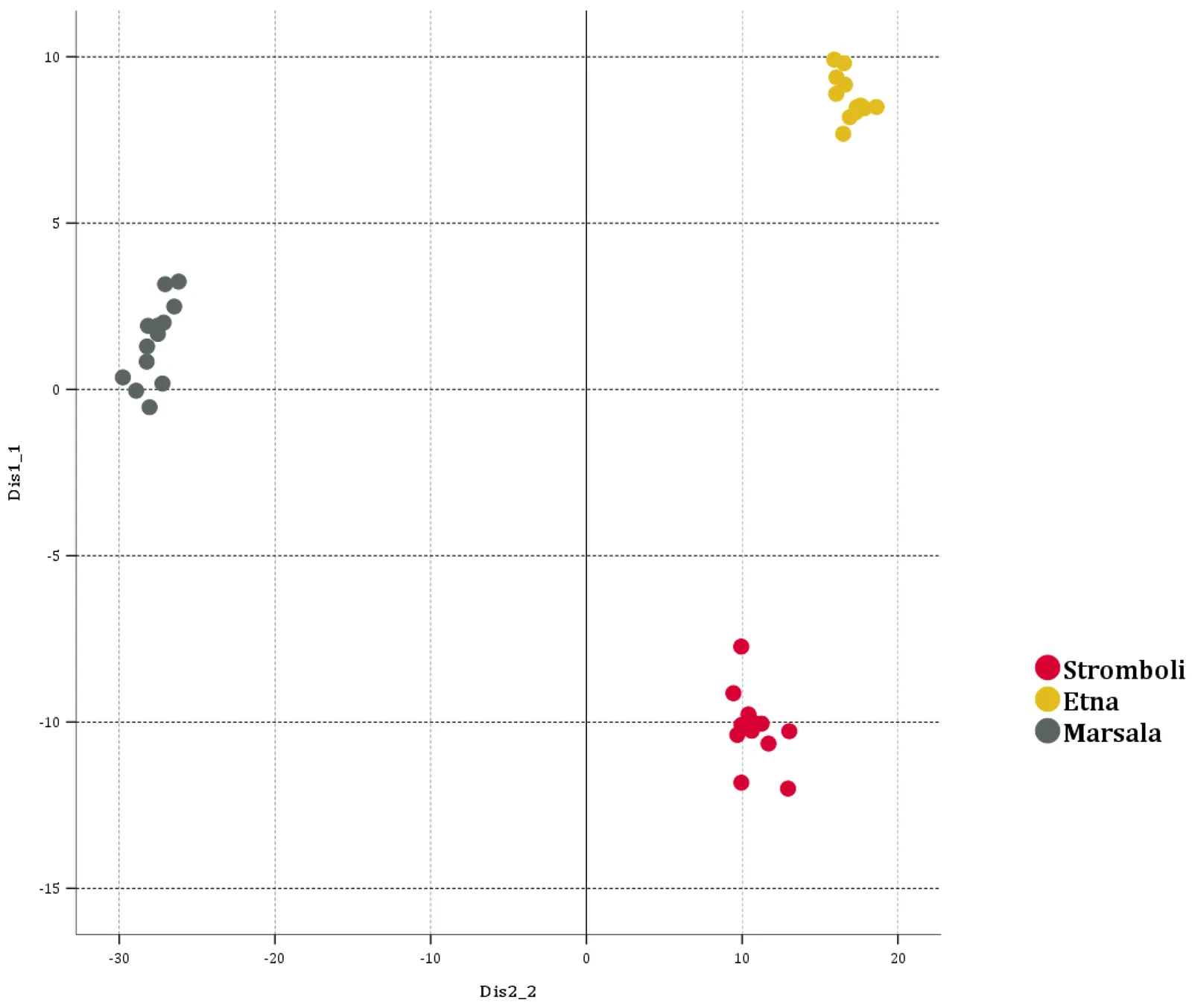
LDA score plot of citrus samples (*n* = 39) according to geographical origin (Stromboli, Etna, Marsala).

Consistent with the separation among the three geographical groups observed in the discriminant space (Figure 4, where the plot displays the distribution of samples along the first two canonical functions), the classification results showed complete classification within the investigated dataset. All samples were correctly assigned to their geographical origin in both the original and leave-one-out cross-validation procedures. Each case was accurately identified even when excluded from the model used for its classification (Table S7 of the Supplementary Materials). Although both the original and leave-one-out cross-validation yielded complete classification within the present dataset, these results should be interpreted with caution because of the relatively limited number of samples. Consequently, the present LDA model should be regarded as a proof-of-concept demonstrating the discriminatory potential of elemental fingerprints rather than a definitive geographical authentication model. This limitation had also been recognized in food authentication research employing chemometric models based on relatively small datasets [59]. Accordingly, external validation using independent harvest years and additional orchards will be necessary to evaluate the generalizability of the proposed model.

### 3.6. Contribution of Juices to Reference Values

Table S8 of the Supplementary Materials reports the percentage contribution of a standard 200 mL serving of various citrus juices (lemon, mandarin, yellow grapefruit, and pink grapefruit) sourced from different geographical origins (Stromboli, Etna, Marsala) to daily Nutrient Reference Values (NRVs), or Toxicological Reference Values (TRVs), for mineral elements under analysis. These percentage contributions were calculated based on the Estimated Daily Intake (EDI), as described in Section 2.9.

For essential mineral elements, a 200 mL serving of the analyzed citrus juices generally provided a modest contribution to the daily NRVs. K showed the highest contributions across all juice types and origins, ranging from 9.92% (pink grapefruit juice from Marsala) to 17.28% (lemon juice from Stromboli) of the NRV. P, Ca, and Fe contributions were generally moderate, with maximum values of 7.52% for P (pink grapefruit juice from Etna), 7.54% for Ca (mandarin juice from Marsala), and 4.37% for Fe (yellow grapefruit juice from Stromboli). Mg contributions were notably higher in some grapefruit varieties, reaching up to 11.53% in yellow grapefruit juice from Marsala and 10.06% in pink grapefruit juice from Etna. Zn contributions also varied, with yellow and pink grapefruit juices generally showing higher contributions, reaching 6.46% in yellow grapefruit juice from Etna. Among trace elements, Cu showed contributions up to 15.20% (pink grapefruit juice from Etna), while Mo showed greater variability, reaching 48.00% in pink grapefruit juice from Etna and 37.20% in yellow grapefruit juice from Stromboli. Se also showed a relatively high contribution, particularly in yellow grapefruit juice from Etna (33.82%). Cr showed its highest contribution in pink grapefruit juice from Stromboli, corresponding to 60.00% of the NRV per 200 mL serving. Na contributions were consistently low across all samples, accounting for less than 0.14% of the adequate intake.

Regarding potentially toxic and toxic elements, concentrations in the analyzed citrus juices were generally low or below the LOQ, resulting in estimated exposures well below the selected toxicological reference values. Ni and Al contributions were low, with maximum values of 0.95% of the TDI for Ni in yellow grapefruit juice from Marsala and 0.49% of the TWI for Al in pink grapefruit juice from Etna. For several elements, including V, Pb, As, Sb, Ti, and Hg, specific percentage contribution values were not provided in the table across all juice types and origins. This is because their concentrations in the samples were below the limit of quantification. Co was quantified in some samples, with the highest contribution being 0.19% of the MRL for mandarin juice from Stromboli. Cd was quantified in lemon juice from Stromboli, with the estimated exposure corresponding to 4.00% of the Tolerable Weekly Intake (TWI).

In summary, the analysis indicated that a 200 mL serving of these Sicilian citrus juices can contribute to the intake of several essential minerals, while the measured concentrations of potentially toxic elements remained low relative to the selected toxicological reference values. Grapefruit juices (yellow and pink), particularly those from Etna and Stromboli, showed the highest contributions for Mo, Se, Cu, Mg, and Cr. Lemon juice from Stromboli showed the highest contribution for K, while mandarin juice from Marsala showed the highest contribution for Ca.

## 4. Conclusions

This study showed that citrus fruits cultivated in the investigated volcanic and non-volcanic areas of Sicily exhibited distinct elemental profiles consistent with differences in the geological characteristics of the cultivation sites. Because other environmental variables were not independently controlled, these findings should be interpreted as evidence of an association rather than a direct causal relationship. Citrus fruits from the investigated volcanic sites generally contained higher concentrations of K, P, Fe, and Al, whereas fruits from Marsala were characterized by higher Ca and Mg concentrations. A discrepancy was identified for Cr and Pb; despite their significantly higher concentrations in Marsala soil, their concentrations in the fruit parts were consistently very low or below the LOQ. This observation indicates that total soil concentrations alone do not explain the levels measured in the fruits, while the specific factors controlling element availability and uptake were not investigated in the present study.

The peel generally showed higher measured elemental concentrations than the edible fractions, while potentially toxic elements remained low in pulp and juice.

PCA revealed distinct clustering among the investigated geographical groups, whereas LDA correctly classified all samples within the investigated dataset, with K, Al, Mg, Ni, Cd, Na, Ca, and V identified as the most influential discriminant variables. Overall, the present findings suggest that elemental fingerprinting combined with chemometric analysis may support the geographical differentiation of citrus fruits cultivated on contrasting geological substrates. These findings suggest the potential of this approach as a complementary tool for geographical traceability and authenticity assessment of high-value citrus products, including those marketed under Protected Designation of Origin (PDO) or Protected Geographical Indication (PGI) schemes, provided that its performance is confirmed using larger independent datasets from additional orchards and harvest years.

## Figures and Tables

**Table 1:** Element concentrations (mean ± SD; mg·kg^−1^) in soil and whole fruit samples from volcanic (Stromboli and Mount Etna) and non-volcanic (Marsala) areas, expressed on an as-is basis.

**Samples**	**Origin**	**K**	**P**	**Ca**	**Mg**	**Na**	**Fe**	**Zn**	**Cu**	**Mn**	**Al**	**Mo**
S	Stromboli (*n* = 4)	19,044 ± 546 ^c^	2238 ± 20 ^b^	6955 ± 956 ^a^	11,272 ± 1111 ^a^	3325 ± 47 ^a^	62,822 ± 1899 ^c^	83.9 ± 109.1 ^c^	60.0 ± 6.3 ^b^	1186 ± 77 ^b^	88,517 ± 313 ^c^	0.78 ± 0.10 ^a^
S	M. Etna (*n* = 4)	13,971 ± 1257 ^b^	2500 ± 41 ^b^	6734 ± 272 ^a^	14,330 ± 261 ^b^	4483 ± 175 ^b^	45,977 ± 986 ^b^	114.6 ±5.4 ^b^	111.9 ± 2.7 ^c^	1500 ± 41 ^b^	66,887 ± 944 ^b^	1.48 ± 0.22 ^b^
S	Marsala (*n* = 4)	6241 ± 183 ^a^	882 ± 94 ^a^	8705 ± 120 ^b^	18,495 ± 364 ^c^	5742 ± 138 ^c^	29,208 ± 1723 ^a^	53.1 ± 10.2 ^a^	31.9 ± 9.3 ^a^	881 ± 95 ^a^	36,542 ± 481 ^a^	0.53 ± 0.05 ^a^
L_WF	Stromboli (*n* = 4)	2026 ± 10 ^b^	242 ± 6 ^b^	247 ± 4 ^b^	131 ± 3 ^b^	20.48 ± 0.40 ^c^	5.44 ± 0.12 ^b^	2.57 ± 0.04 ^a^	0.56 ± 0.01 ^b^	0.37 ± 0.01 ^b^	0.254 ± 0.005 ^b^	0.051 ± 0.002 ^b^
L_WF	M. Etna (*n* = 4)	1871 ± 12 ^b^	234 ± 3 ^b^	180 ± 3 ^a^	87 ± 1 ^a^	7.71 ± 0.57 ^a^	4.77 ± 0.05 ^b^	2.34 ± 0.13 ^a^	0.50 ± 0.03 ^b^	0.47 ± 0.01 ^b^	0.241 ± 0.011 ^b^	0.094 ± 0.010 ^c^
L_WF	Marsala (*n* = 4)	1642 ± 13 ^a^	173 ± 5 ^a^	335 ± 4 ^c^	150 ± 3 ^b^	15.35 ± 0.61 ^b^	4.00 ± 0.04 ^a^	1.95 ± 0.06 ^a^	0.36 ± 0.02 ^a^	0.20 ± 0.01 ^a^	0.198 ± 0.018 ^a^	0.026 ± 0.002 ^a^
M_WF	Stromboli (*n* = 3)	1672 ± 1 ^b^	243 ± 3 ^b^	282 ± 2 ^a^	120 ± 1 ^b^	17.79 ± 0.65 ^b^	2.95 ± 0.10 ^b^	1.80 ± 0.03 ^a^	0.62 ± 0.01 ^a^	0.74 ± 0.01 ^b^	0.116 ± 0.001 ^b^	0.077 ± 0.004 ^ab^
M_WF	M. Etna (*n* = 3)	1460 ± 35 ^a^	255 ± 12 ^b^	236 ± 2 ^a^	92 ± 2 ^a^	10.57 ± 0.20 ^a^	1.93 ± 0.16 ^a^	1.75 ± 0.12 ^a^	0.90 ± 0.01 ^c^	0.36 ± 0.03 ^a^	0.135 ± 0.004 ^c^	0.090 ± 0.003 ^b^
M_WF	Marsala (*n* = 3)	1487 ± 5 ^a^	168 ± 2 ^a^	374 ± 1 ^b^	149 ± 1 ^b^	12.16 ± 0.29 ^a^	2.16 ± 0.01 ^a^	1.38 ± 0.02 ^a^	0.38 ± 0.02 ^a^	0.51 ± 0.02 ^ab^	0.072 ± 0.002 ^a^	0.049 ± 0.003 ^a^
YG_WF	Stromboli (*n* = 3)	1616 ± 11 ^b^	275 ± 2 ^b^	314 ± 2 ^b^	215 ± 2 ^b^	14.15 ± 0.20 ^b^	4.07 ± 0.07 ^b^	2.70 ± 0.02 ^a^	0.59 ± 0.01 ^b^	0.55 ± 0.02 ^b^	0.239 ± 0.011 ^b^	0.147 ± 0.016 ^b^
YG_WF	M. Etna (*n* = 3)	1580 ± 12 ^b^	285 ± 1 ^b^	239 ± 1 ^a^	108 ± 2 ^a^	12.77 ± 0.48 ^b^	1.20 ± 0.15 ^a^	4.05 ± 0.10 ^b^	0.87 ± 0.02 ^c^	0.53 ± 0.02 ^b^	0.300 ± 0.046 ^c^	0.119 ± 0.003 ^b^
YG_WF	Marsala (*n* = 3)	1168 ± 3 ^a^	150 ± 3 ^a^	422 ± 3 ^c^	245 ± 2 ^b^	7.76 ± 0.12 ^a^	2.32 ± 0.03 ^a^	2.17 ± 0.03 ^a^	0.39 ± 0.00 ^a^	0.42 ± 0.01 ^a^	0.101 ± 0.011 ^a^	0.031 ± 0.002 ^a^
PG_WF	Stromboli (*n* = 3)	1481 ± 5 ^b^	268 ± 1 ^b^	290 ± 4 ^b^	186 ± 1 ^b^	10.44 ± 0.47 ^b^	3.29 ± 0.09 ^b^	2.64 ± 0.02 ^a^	0.71 ± 0.02 ^b^	0.51 ± 0.02 ^b^	0.329 ± 0.019 ^c^	0.105 ± 0.005 ^b^
PG_WF	M. Etna (*n* = 3)	1603 ± 17 ^b^	306 ± 3 ^b^	320 ± 4 ^b^	213 ± 15 ^b^	12.44 ± 0.34 ^b^	3.75 ± 0.07 ^b^	2.92 ± 0.05 ^a^	0.94 ± 0.02 ^b^	0.60 ± 0.04 ^b^	0.404 ± 0.013 ^b^	0.190 ± 0.007 ^b^
PG_WF	Marsala (*n* = 3)	1048 ± 3 ^a^	140 ± 2 ^a^	217 ± 1 ^a^	94 ± 1 ^a^	6.93 ± 0.13 ^a^	1.99 ± 0.03 ^a^	2.11 ± 0.03 ^a^	0.55 ± 0.00 ^a^	0.27 ± 0.01 ^a^	0.071 ± 0.002 ^a^	0.024 ± 0.006 ^a^
**Samples**	**Origin**	**Se**	**Ni**	**Cr**	**V**	**Co**	**Pb**	**As**	**Sb**	**Ti**	**Cd**	**Hg**
S	Stromboli (*n* = 4)	0.48 ± 0.13 ^a^	17.20 ± 1.81 ^a^	2.68 ± 0.95 ^a^	60.05 ± 9.97 ^a^	13.78 ± 0.56 ^b^	11.65 ± 0.51 ^a^	4.50 ± 0.78 ^a^	2.05 ± 0.31 ^a^	1.93 ± 0.31 ^b^	0.85 ± 0.21 ^a^	0.048 ± 0.013 ^a^
S	M. Etna (*n* = 4)	4.40 ± 0.51 ^b^	35.80 ± 1.66 ^b^	13.30 ± 1.32 ^b^	84.25 ± 3.46 ^b^	18.00 ± 1.47 ^b^	41.40 ± 3.06 ^b^	5.78 ± 0.25 ^a^	2.35 ± 0.24 ^a^	1.20 ± 0.26 ^b^	2.00 ± 0.44 ^b^	0.058 ± 0.005 ^a^
S	Marsala (*n* = 4)	0.28 ± 0.10 ^a^	42.38 ± 1.60 ^b^	48.95 ± 5.55 ^c^	60.18 ± 3.59 ^a^	7.90 ± 0.43 ^a^	75.35 ± 7.87 ^c^	8.00 ± 0.36 ^b^	2.88 ± 0.31 ^a^	0.60 ± 0.14 ^a^	0.63 ± 0.13 ^a^	0.023 ± 0.005 ^a^
L_WF	Stromboli (*n* = 4)	0.054 ± 0.004 ^b^	0.049 ± 0.004 ^b^	0.057 ± 0.004 ^b^	0.014 ± 0.002 ^a^	0.012 ± 0.002 ^a^	0.014 ± 0.002 ^a^	0.006 ± 0.002 ^a^	0.008 ± 0.002 ^a^	0.010 ± 0.000 ^a^	0.006 ± 0.000 ^a^	0.005 ± 0.000 ^a^
L_WF	M. Etna (*n* = 4)	0.051 ± 0.007 ^b^	0.051 ± 0.005 ^b^	0.064 ± 0.002 ^b^	0.016 ± 0.004 ^a^	0.018 ± 0.001 ^a^	0.022 ± 0.002 ^a^	0.011 ± 0.001 ^a^	0.010 ± 0.002 ^a^	0.012 ± 0.003 ^a^	0.021 ± 0.004 ^b^	0.006 ± 0.001 ^a^
L_WF	Marsala (*n* = 4)	0.024 ± 0.003 ^a^	0.030 ± 0.004 ^a^	0.034 ± 0.007 ^a^	<LOQ	<LOQ	<LOQ	<LOQ	<LOQ	<LOQ	<LOQ	<LOQ
M_WF	Stromboli (*n* = 3)	0.035 ± 0.004 ^b^	0.025 ± 0.002 ^b^	0.026 ± 0.002 ^a^	0.012 ± 0.001 ^b^	0.004 ± 0.001 ^a^	0.003 ± 0.000	0.004 ± 0.000 ^a^	0.003 ± 0.000 ^a^	0.008 ± 0.001 ^a^	0.002 ± 0.001 ^a^	0.006 ± 0.001 ^a^
M_WF	M. Etna (*n* = 3)	0.040 ± 0.001 ^b^	0.034 ± 0.004 ^b^	0.043 ± 0.008 ^b^	0.017 ± 0.002 ^b^	0.009 ± 0.003 ^a^	0.004 ± 0.001	0.006 ± 0.000 ^a^	0.003 ± 0.000 ^a^	0.011 ± 0.003 ^a^	0.003 ± 0.001 ^a^	0.007 ± 0.001 ^a^
M_WF	Marsala (*n* = 3)	0.017 ± 0.001 ^a^	0.017 ± 0.002 ^a^	0.014 ± 0.001 ^a^	0.003 ± 0.001 ^a^	<LOQ	0.002 ± 0.001	<LOQ	<LOQ	<LOQ	<LOQ	<LOQ
YG_WF	Stromboli (*n* = 3)	0.066 ± 0.004 ^b^	0.052 ± 0.007 ^b^	0.084 ± 0.003 ^b^	0.014 ± 0.000 ^b^	0.029 ± 0.002 ^a^	0.008 ± 0.000 ^b^	0.007 ± 0.002 ^a^	0.004 ± 0.000 ^a^	0.011 ± 0.000 ^a^	0.013 ± 0.001 ^a^	0.005 ± 0.000 ^a^
YG_WF	M. Etna (*n* = 3)	0.131 ± 0.003 ^c^	0.045 ± 0.003 ^b^	0.047 ± 0.004 ^a^	0.030 ± 0.009 ^c^	0.038 ± 0.003 ^b^	0.010 ± 0.003 ^b^	0.013 ± 0.002 ^a^	0.008 ± 0.002 ^a^	0.018 ± 0.006 ^a^	0.020 ± 0.004 ^b^	0.003 ± 0.001 ^a^
YG_WF	Marsala (*n* = 3)	0.024 ± 0.002 ^a^	0.010 ± 0.002 ^a^	0.036 ± 0.005 ^a^	0.003 ± 0.000 ^a^	<LOQ	0.003 ± 0.001 ^a^	<LOQ	<LOQ	<LOQ	<LOQ	<LOQ
PG_WF	Stromboli (*n* = 3)	0.050 ± 0.002 ^b^	0.038 ± 0.002 ^b^	0.132 ± 0.010 ^b^	0.009 ± 0.002 ^a^	0.020 ± 0.003 ^a^	0.014 ± 0.001 ^b^	0.005 ± 0.002 ^a^	0.005 ± 0.002 ^a^	0.018 ± 0.003 ^a^	0.008 ± 0.002 ^a^	0.005 ± 0.002 ^a^
PG_WF	M. Etna (*n* = 3)	0.081 ± 0.001 ^c^	0.094 ± 0.007 ^c^	0.231 ± 0.011 ^c^	0.019 ± 0.003 ^b^	0.036 ± 0.006 ^b^	0.015 ± 0.002 ^b^	0.003 ± 0.000 ^a^	0.005 ± 0.002 ^a^	0.027 ± 0.002 ^b^	0.022 ± 0.005 ^b^	0.011 ± 0.005 ^a^
PG_WF	Marsala (*n* = 3)	0.021 ± 0.003 ^a^	0.004 ± 0.002 ^a^	0.040 ± 0.004 ^a^	0.004 ± 0.002 ^a^	<LOQ	0.004 ± 0.002 ^a^	<LOQ	<LOQ	<LOQ	<LOQ	<LOQ

S, Soil; L, Lemon; M, Mandarin; YG, Yellow Grapefruit; PG, Pink Grapefruit; WF, Whole Fruit; Superscript letters (a, b, c) indicate statistically significant differences (*p* < 0.05) among samples from Stromboli, Etna, and Marsala.

**Table 2:** Element concentrations (mean ± SD; mg·kg^−1^ for pulp and peel; mg·L^−1^ for juice) in citrus fruit fractions from volcanic (Stromboli and Mount Etna) and non-volcanic (Marsala) areas, expressed on an as-is basis.

**Samples**	**Origin**	**K**	**P**	**Ca**	**Mg**	**Na**	**Fe**	**Zn**	**Cu**	**Mn**	**Al**	**Mo**
L_J	Stromboli (*n* = 4)	1728 ± 18 ^c^	172 ± 6 ^b^	117 ± 6 ^b^	69 ± 3 ^b^	9.28 ± 0.22 ^b^	2.28 ± 0.10 ^b^	0.66 ± 0.05 ^a^	0.33 ± 0.02 ^b^	0.22 ± 0.02 ^b^	0.058 ± 0.010 ^b^	0.030 ± 0.004 ^b^
L_J	M. Etna (*n* = 4)	1556 ± 18 ^b^	170 ± 5 ^b^	87 ± 2 ^a^	46 ± 2 ^a^	5.33 ± 0.82 ^a^	1.92 ± 0.08 ^ab^	0.81 ± 0.11 ^b^	0.24 ± 0.03 ^a^	0.26 ± 0.02 ^b^	0.073 ± 0.010 ^b^	0.048 ± 0.005 ^b^
L_J	Marsala (*n* = 4)	1380 ± 27 ^a^	108 ± 2 ^a^	163 ± 5 ^c^	77 ± 3 ^b^	6.97 ± 0.23 ^ab^	1.49 ± 0.07 ^a^	0.50 ± 0.03 ^a^	0.23 ± 0.02 ^a^	0.10 ± 0.01 ^a^	0.038 ± 0.005 ^a^	0.010 ± 0.005 ^a^
L_Pu	Stromboli (*n* = 4)	2047 ± 18 ^b^	246 ± 6 ^b^	258 ± 5 ^b^	144 ± 6 ^b^	21.01 ± 1.23 ^c^	5.74 ± 0.18 ^b^	3.51 ± 0.13 ^b^	0.64 ± 0.03 ^b^	0.54 ± 0.03 ^b^	0.338 ± 0.021 ^b^	0.053 ± 0.005 ^b^
L_Pu	M. Etna (*n* = 4)	1967 ± 24 ^b^	240 ± 8 ^b^	194 ± 3 ^a^	100 ± 2 ^a^	7.95 ± 0.47 ^a^	4.91 ± 0.10 ^ab^	2.70 ± 0.37 ^a^	0.59 ± 0.05 ^b^	0.65 ± 0.02 ^b^	0.300 ± 0.048 ^b^	0.093 ± 0.019 ^c^
L_Pu	Marsala (*n* = 4)	1750 ± 31 ^a^	185 ± 3 ^a^	358 ± 4 ^c^	163 ± 5 ^b^	14.16 ± 0.21 ^b^	4.66 ± 0.07 ^a^	2.44 ± 0.09 ^a^	0.40 ± 0.02 ^a^	0.35 ± 0.03 ^a^	0.218 ± 0.010 ^a^	0.020 ± 0.003 ^a^
L_Pe	Stromboli (*n* = 4)	2353 ± 37 ^c^	318 ± 13 ^b^	389 ± 8 ^b^	193 ± 6 ^b^	32.91 ± 1.72 ^c^	8.84 ± 0.20 ^b^	4.08 ± 0.06 ^a^	0.76 ± 0.03 ^b^	0.43 ± 0.02 ^b^	0.420 ± 0.012 ^a^	0.073 ± 0.010 ^a^
L_Pe	M. Etna (*n* = 4)	2162 ± 41 ^b^	302 ± 7 ^b^	275 ± 4 ^a^	125 ± 2 ^a^	10.25 ± 0.73 ^a^	7.93 ± 0.07 ^b^	3.83 ± 0.27 ^ab^	0.74 ± 0.04 ^b^	0.59 ± 0.03 ^b^	0.393 ± 0.013 ^a^	0.148 ± 0.019 ^b^
L_Pe	Marsala (*n* = 4)	1866 ± 28 ^a^	240 ± 13 ^a^	514 ± 10 ^c^	225 ± 5 ^b^	25.77 ± 1.48 ^b^	6.38 ± 0.13 ^a^	3.26 ± 0.14 ^a^	0.48 ± 0.05 ^a^	0.23 ± 0.03 ^a^	0.368 ± 0.046 ^a^	0.048 ± 0.005 ^a^
M_J	Stromboli (*n* = 3)	1569 ± 14 ^b^	199 ± 2 ^b^	231 ± 3 ^b^	80 ± 2 ^a^	13.72 ± 0.43 ^b^	2.05 ± 0.10 ^b^	0.66 ± 0.05 ^b^	0.43 ± 0.01 ^b^	0.52 ± 0.03 ^b^	0.053 ± 0.006 ^b^	0.060 ± 0.010 ^ab^
M_J	M. Etna (*n* = 3)	1359 ± 35 ^a^	214 ± 16 ^b^	189 ± 6 ^a^	71 ± 2 ^a^	9.97 ± 0.65 ^a^	1.11 ± 0.13 ^a^	0.73 ± 0.17 ^b^	0.62 ± 0.03 ^b^	0.22 ± 0.02 ^a^	0.063 ± 0.012 ^b^	0.073 ± 0.006 ^b^
M_J	Marsala (*n* = 3)	1367 ± 11 ^a^	138 ± 2 ^a^	302 ± 3 ^c^	103 ± 2 ^b^	9.72 ± 0.30 ^a^	1.49 ± 0.04 ^a^	0.33 ± 0.02 ^a^	0.22 ± 0.02 ^a^	0.32 ± 0.02 ^ab^	0.023 ± 0.006 ^a^	0.033 ± 0.006 ^a^
M_Pu	Stromboli (*n* = 3)	1736 ± 16 ^b^	277 ± 4 ^b^	293 ± 4 ^a^	166 ± 1 ^ab^	24.48 ± 1.03 ^c^	3.40 ± 0.16 ^b^	2.75 ± 0.07 ^b^	0.73 ± 0.03 ^b^	0.86 ± 0.06 ^b^	0.157 ± 0.006 ^b^	0.080 ± 0.010 ^ab^
M_Pu	M. Etna (*n* = 3)	1483 ± 139 ^a^	271 ± 16 ^b^	252 ± 5 ^a^	103 ± 2 ^a^	9.40 ± 0.68 ^a^	1.78 ± 0.31 ^a^	2.65 ± 0.12 ^b^	0.93 ± 0.16 ^b^	0.47 ± 0.20 ^a^	0.180 ± 0.010 ^b^	0.100 ± 0.010 ^b^
M_Pu	Marsala (*n* = 3)	1554 ± 24 ^a^	192 ± 2 ^a^	396 ± 5 ^b^	203 ± 2 ^b^	15.98 ± 0.25 ^b^	2.36 ± 0.08 ^ab^	2.22 ± 0.04 ^a^	0.50 ± 0.02 ^a^	0.53 ± 0.03 ^a^	0.107 ± 0.015 ^a^	0.057 ± 0.006 ^a^
M_Pe	Stromboli (*n* = 3)	1849 ± 13 ^b^	313 ± 8 ^b^	385 ± 5 ^a^	170 ± 1 ^ab^	21.40 ± 1.08 ^b^	4.55 ± 0.13 ^b^	3.55 ± 0.09	0.95 ± 0.03 ^ab^	1.13 ± 0.07 ^b^	0.220 ± 0.010 ^b^	0.110 ± 0.010 ^b^
M_Pe	M. Etna (*n* = 3)	1662 ± 36 ^a^	333 ± 12 ^b^	326 ± 9 ^a^	130 ± 4 ^a^	12.83 ± 0.64 ^a^	3.85 ± 0.25 ^a^	3.26 ± 0.07	1.48 ± 0.12 ^b^	0.57 ± 0.07 ^a^	0.257 ± 0.006 ^b^	0.120 ± 0.010 ^b^
M_Pe	Marsala (*n* = 3)	1695 ± 7 ^a^	214 ± 4 ^a^	515 ± 4 ^b^	206 ± 1 ^b^	14.49 ± 0.34 ^a^	3.49 ± 0.07 ^a^	3.03 ± 0.07	0.63 ± 0.02 ^a^	0.90 ± 0.04 ^ab^	0.150 ± 0.010 ^a^	0.077 ± 0.006 ^a^
YG_J	Stromboli (*n* = 3)	1468 ± 11 ^b^	249 ± 3 ^b^	205 ± 2 ^a^	195 ± 4 ^b^	11.19 ± 0.39 ^b^	3.06 ± 0.04 ^b^	2.43 ± 0.05 ^ab^	0.46 ± 0.02	0.31 ± 0.04 ^b^	0.110 ± 0.010 ^b^	0.093 ± 0.015 ^b^
YG_J	M. Etna (*n* = 3)	1435 ± 19 ^b^	260 ± 5 ^b^	163 ± 3 ^a^	81 ± 2 ^a^	10.46 ± 0.85 ^b^	0.72 ± 0.15 ^a^	3.23 ± 0.10 ^b^	0.54 ± 0.01	0.32 ± 0.06 ^b^	0.127 ± 0.021 ^b^	0.067 ± 0.012 ^b^
YG_J	Marsala (*n* = 3)	1009 ± 4 ^a^	119 ± 3 ^a^	286 ± 2 ^b^	216 ± 4 ^b^	5.38 ± 0.19 ^a^	1.66 ± 0.06 ^a^	1.89 ± 0.04 ^a^	0.30 ± 0.01	0.19 ± 0.02 ^a^	0.043 ± 0.006 ^a^	0.010 ± 0.002 ^a^
YG_Pu	Stromboli (*n* = 3)	1720 ± 6 ^c^	297 ± 3 ^b^	328 ± 3 ^b^	214 ± 2 ^b^	15.34 ± 0.07 ^b^	4.10 ± 0.07 ^c^	2.80 ± 0.04 ^a^	0.60 ± 0.03 ^ab^	0.45 ± 0.04 ^b^	0.267 ± 0.021 ^b^	0.167 ± 0.015 ^b^
YG_Pu	M. Etna (*n* = 3)	1564 ± 110 ^b^	301 ± 4 ^b^	224 ± 4 ^a^	110 ± 1 ^a^	12.27 ± 0.27 ^b^	1.12 ± 0.11 ^a^	3.86 ± 0.17 ^b^	0.91 ± 0.06 ^b^	0.41 ± 0.03 ^b^	0.357 ± 0.031 ^b^	0.120 ± 0.010 ^b^
YG_Pu	Marsala (*n* = 3)	1275 ± 6 ^a^	170 ± 4 ^a^	438 ± 3 ^c^	250 ± 3 ^b^	9.01 ± 0.23 ^a^	2.81 ± 0.06 ^b^	2.38 ± 0.04 ^a^	0.43 ± 0.01 ^a^	0.33 ± 0.02 ^a^	0.117 ± 0.015 ^a^	0.033 ± 0.006 ^a^
YG_Pe	Stromboli (*n* = 3)	1796 ± 16 ^b^	305 ± 2 ^b^	484 ± 4 ^b^	249 ± 3 ^b^	18.30 ± 0.02 ^b^	5.72 ± 0.12 ^c^	3.07 ± 0.07 ^ab^	0.80 ± 0.04 ^a^	1.03 ± 0.03 ^b^	0.437 ± 0.025 ^b^	0.223 ± 0.023 ^b^
YG_Pe	M. Etna (*n* = 3)	1831 ± 7 ^b^	317 ± 7 ^b^	374 ± 3 ^a^	153 ± 4 ^a^	16.95 ± 0.55 ^b^	2.04 ± 0.25 ^a^	5.52 ± 0.34 ^b^	1.38 ± 0.04 ^b^	0.94 ± 0.03 ^ab^	0.550 ± 0.100 ^b^	0.207 ± 0.015 ^b^
YG_Pe	Marsala (*n* = 3)	1363 ± 12 ^a^	189 ± 3 ^a^	637 ± 7 ^c^	291 ± 4 ^b^	10.89 ± 0.30 ^a^	3.10 ± 0.05 ^b^	2.51 ± 0.04 ^a^	0.52 ± 0.02 ^a^	0.87 ± 0.06 ^a^	0.187 ± 0.021 ^a^	0.063 ± 0.006 ^a^
PG_J	Stromboli (*n* = 3)	1326 ± 5 ^b^	232 ± 1 ^b^	193 ± 3 ^ab^	174 ± 2 ^b^	8.61 ± 0.50 ^b^	2.74 ± 0.17 ^b^	2.15 ± 0.07 ^b^	0.58 ± 0.03 ^ab^	0.19 ± 0.03 ^b^	0.163 ± 0.012 ^b^	0.053 ± 0.006 ^a^
PG_J	M. Etna (*n* = 3)	1479 ± 3 ^b^	263 ± 4 ^b^	225 ± 2 ^b^	189 ± 3 ^b^	9.70 ± 0.62 ^b^	3.04 ± 0.14 ^b^	2.41 ± 0.03 ^b^	0.76 ± 0.05 ^b^	0.27 ± 0.05 ^b^	0.247 ± 0.015 ^c^	0.120 ± 0.010 ^b^
PG_J	Marsala (*n* = 3)	992 ± 4 ^a^	102 ± 2 ^a^	145 ± 3 ^a^	68 ± 2 ^a^	4.63 ± 0.17 ^a^	1.49 ± 0.051 ^a^	1.69 ± 0.05 ^a^	0.43 ± 0.02 ^a^	0.03 ± 0.01 ^a^	0.033 ± 0.006 ^a^	0.013 ± 0.006 ^a^
PG_Pu	Stromboli (*n* = 3)	1550 ± 34 ^b^	283 ± 5 ^b^	289 ± 2 ^b^	183 ± 2 ^b^	11.75 ± 0.27 ^b^	3.80 ± 0.10 ^b^	2.63 ± 0.08	0.70 ± 0.03 ^ab^	0.29 ± 0.04 ^b^	0.387 ± 0.025 ^b^	0.103 ± 0.021 ^b^
PG_Pu	M. Etna (*n* = 3)	1644 ± 49 ^b^	335 ± 5 ^b^	330 ± 5 ^b^	202 ± 2 ^b^	13.09 ± 0.13 ^b^	4.14 ± 0.07 ^b^	2.82 ± 0.07	0.97 ± 0.07 ^b^	0.41 ± 0.03 ^b^	0.450 ± 0.030 ^b^	0.207 ± 0.015 ^c^
PG_Pu	Marsala (*n* = 3)	1023 ± 8 ^a^	158 ± 3 ^a^	206 ± 6 ^a^	100 ± 1 ^a^	8.59 ± 0.33 ^a^	2.22 ± 0.10 ^a^	2.23 ± 0.05	0.54 ± 0.02 ^a^	0.13 ± 0.01 ^a^	0.083 ± 0.006 ^a^	0.023 ± 0.006 ^a^
PG_Pe	Stromboli (*n* = 3)	1694 ± 8 ^b^	317 ± 4 ^b^	452 ± 7 ^b^	208.8 ± 4.1 ^b^	12.62 ± 0.54 ^b^	3.86 ± 0.10 ^b^	3.47 ± 0.08 ^b^	0.94 ± 0.02 ^a^	1.19 ± 0.04 ^b^	0.567 ± 0.040 ^b^	0.193 ± 0.025 ^b^
PG_Pe	M. Etna (*n* = 3)	1780 ± 21 ^b^	357 ± 5 ^b^	472 ± 12 ^b^	261.0 ± 55.1 ^b^	16.57 ± 0.14 ^b^	4.67 ± 0.10 ^b^	3.85 ± 0.09 ^b^	1.22 ± 0.05 ^b^	1.28 ± 0.06 ^b^	0.637 ± 0.042 ^b^	0.297 ± 0.031 ^c^
PG_Pe	Marsala (*n* = 3)	1157 ± 18 ^a^	190 ± 8 ^a^	343 ± 3 ^a^	131.9 ± 1.4 ^a^	9.66 ± 0.12 ^a^	2.66 ± 0.10 ^a^	2.73 ± 0.09 ^a^	0.73 ± 0.03 ^a^	0.77 ± 0.02 ^a^	0.127 ± 0.015 ^a^	0.043 ± 0.006 ^a^
**Samples**	**Origin**	**Se**	**Ni**	**Cr**	**V**	**Co**	**Pb**	**As**	**Sb**	**Ti**	**Cd**	**Hg**
L_J	Stromboli (*n* = 4)	0.015 ± 0.006 ^b^	0.010 ± 0.001 ^b^	0.008 ± 0.002 ^a^	<LOQ	<LOQ	<LOQ	<LOQ	<LOQ	<LOQ	<LOQ	<LOQ
L_J	M. Etna (*n* = 4)	0.013 ± 0.005 ^b^	0.023 ± 0.005 ^c^	0.011 ± 0.004 ^a^	<LOQ	<LOQ	<LOQ	<LOQ	<LOQ	<LOQ	<LOQ	<LOQ
L_J	Marsala (*n* = 4)	0.005 ± 0.001 ^a^	0.004 ± 0.001 ^a^	<LOQ	<LOQ	<LOQ	<LOQ	<LOQ	<LOQ	<LOQ	<LOQ	<LOQ
L_Pu	Stromboli (*n* = 4)	0.053 ± 0.005 ^b^	0.055 ± 0.006 ^c^	0.070 ± 0.008 ^b^	0.010 ± 0.001 ^a^	0.010 ± 0.001 ^a^	0.010 ± 0.001 ^a^	<LOQ	<LOQ	0.010 ± 0.001 ^a^	0.007 ± 0.001 ^a^	<LOQ
L_Pu	M. Etna (*n* = 4)	0.058 ± 0.005 ^b^	0.043 ± 0.005 ^b^	0.073 ± 0.009 ^b^	0.013 ± 0.005 ^a^	0.025 ± 0.002 ^a^	0.013 ± 0.005 ^a^	0.013 ± 0.001	<LOQ	0.013 ± 0.005 ^a^	0.015 ± 0.004 ^b^	<LOQ
L_Pu	Marsala (*n* = 4)	0.020 ± 0.003 ^a^	0.033 ± 0.005 ^a^	0.045 ± 0.010 ^a^	<LOQ	<LOQ	<LOQ	<LOQ	<LOQ	<LOQ	<LOQ	<LOQ
L_Pe	Stromboli (*n* = 4)	0.100 ± 0.008 ^b^	0.090 ± 0.014 ^b^	0.103 ± 0.010 ^b^	0.033 ± 0.005 ^a^	0.026 ± 0.006 ^a^	0.033 ± 0.005 ^a^	0.013 ± 0.005 ^a^	0.018 ± 0.005 ^a^	0.020 ± 0.003 ^a^	0.012 ± 0.001 ^a^	0.012 ± 0.001 ^a^
L_Pe	M. Etna (*n* = 4)	0.090 ± 0.014 ^b^	0.090 ± 0.008 ^b^	0.119 ± 0.011 ^b^	0.038 ± 0.010 ^a^	0.033 ± 0.003 ^a^	0.053 ± 0.010 ^a^	0.022 ± 0.003 ^a^	0.025 ± 0.006 ^a^	0.025 ± 0.010 ^a^	0.049 ± 0.010 ^b^	0.014 ± 0.003 ^a^
L_Pe	Marsala (*n* = 4)	0.048 ± 0.010 ^a^	0.058 ± 0.010 ^a^	0.065 ± 0.013 ^a^	<LOQ	<LOQ	<LOQ	<LOQ	<LOQ	<LOQ	<LOQ	<LOQ
M_J	Stromboli (*n* = 3)	0.017 ± 0.006 ^a^	0.005 ± 0.002 ^a^	0.010 ± 0.002 ^a^	<LOQ	<LOQ	<LOQ	<LOQ	<LOQ	<LOQ	<LOQ	<LOQ
M_J	M. Etna (*n* = 3)	0.020 ± 0.003 ^a^	0.008 ± 0.002 ^a^	0.008 ± 0.004 ^a^	<LOQ	<LOQ	<LOQ	<LOQ	<LOQ	<LOQ	<LOQ	<LOQ
M_J	Marsala (*n* = 3)	<LOQ	<LOQ	<LOQ	<LOQ	<LOQ	<LOQ	<LOQ	<LOQ	<LOQ	<LOQ	<LOQ
M_Pu	Stromboli (*n* = 3)	0.047 ± 0.006 ^b^	0.020 ± 0.003 ^b^	0.033 ± 0.006 ^b^	0.007 ± 0.003 ^a^	<LOQ	<LOQ	<LOQ	<LOQ	0.010 ± 0.002 ^a^	<LOQ	0.006 ± 0.001 ^a^
M_Pu	M. Etna (*n* = 3)	0.053 ± 0.006 ^b^	0.026 ± 0.004 ^b^	0.065 ± 0.009 ^c^	0.008 ± 0.002 ^a^	<LOQ	<LOQ	0.008 ± 0.001	<LOQ	0.017 ± 0.006 ^a^	<LOQ	0.007 ± 0.001 ^a^
M_Pu	Marsala (*n* = 3)	0.020 ± 0.003 ^a^	0.013 ± 0.006 ^a^	0.013 ± 0.006 ^a^	<LOQ	<LOQ	<LOQ	<LOQ	<LOQ	<LOQ	<LOQ	<LOQ
M_Pe	Stromboli (*n* = 3)	0.067 ± 0.006 ^b^	0.073 ± 0.006 ^c^	0.057 ± 0.006 ^a^	0.043 ± 0.006 ^b^	0.013 ± 0.006 ^a^	0.010 ± 0.002 ^a^	0.012 ± 0.001 ^a^	0.010 ± 0.002 ^a^	0.023 ± 0.006 ^a^	0.008 ± 0.003 ^a^	0.018 ± 0.003 ^a^
M_Pe	M. Etna (*n* = 3)	0.073 ± 0.006 ^b^	0.097 ± 0.012 ^b^	0.102 ± 0.018 ^b^	0.060 ± 0.010 ^b^	0.033 ± 0.012 ^a^	0.013 ± 0.006 ^a^	0.015 ± 0.001 ^a^	0.010 ± 0.001 ^a^	0.030 ± 0.010 ^a^	0.012 ± 0.003 ^b^	0.020 ± 0.003 ^a^
M_Pe	Marsala (*n* = 3)	0.040 ± 0.003 ^a^	0.057 ± 0.006 ^a^	0.033 ± 0.006 ^a^	0.010 ± 0.002 ^a^	<LOQ	<LOQ	<LOQ	<LOQ	<LOQ	<LOQ	<LOQ
YG_J	Stromboli (*n* = 3)	0.047 ± 0.006 ^b^	0.017 ± 0.006 ^a^	0.030 ± 0.005 ^b^	<LOQ	0.010 ± 0.003 ^a^	<LOQ	<LOQ	<LOQ	<LOQ	<LOQ	<LOQ
YG_J	M. Etna (*n* = 3)	0.093 ± 0.015 ^c^	0.013 ± 0.006 ^a^	0.017 ± 0.002 ^a^	<LOQ	0.010 ± 0.004 ^a^	<LOQ	<LOQ	<LOQ	<LOQ	<LOQ	<LOQ
YG_J	Marsala (*n* = 3)	0.010 ± 0.003 ^a^	<LOQ	0.010 ± 0.003 ^a^	<LOQ	<LOQ	<LOQ	<LOQ	<LOQ	<LOQ	<LOQ	<LOQ
YG_Pu	Stromboli (*n* = 3)	0.077 ± 0.006 ^a^	0.073 ± 0.006 ^b^	0.117 ± 0.006 ^b^	0.010 ± 0.001 ^a^	0.037 ± 0.006 ^a^	0.010 ± 0.002 ^a^	0.006 ± 0.001 ^a^	<LOQ	0.010 ± 0.002 ^a^	0.011 ± 0.001 ^a^	0.007 ± 0.001 ^a^
YG_Pu	M. Etna (*n* = 3)	0.143 ± 0.021 ^b^	0.063 ± 0.006 ^b^	0.056 ± 0.012 ^a^	0.027 ± 0.015 ^a^	0.033 ± 0.006 ^a^	0.013 ± 0.006 ^a^	0.007 ± 0.001 ^a^	<LOQ	0.017 ± 0.006 ^a^	0.013 ± 0.002 ^b^	0.004 ± 0.002 ^a^
YG_Pu	Marsala (*n* = 3)	0.030 ± 0.005 ^a^	0.010 ± 0.001 ^a^	0.050 ± 0.010 ^a^	<LOQ	<LOQ	<LOQ	<LOQ	<LOQ	<LOQ	<LOQ	<LOQ
YG_Pe	Stromboli (*n* = 3)	0.090 ± 0.006 ^b^	0.097 ± 0.012 ^b^	0.153 ± 0.006 ^b^	0.040 ± 0.006 ^b^	0.057 ± 0.006 ^a^	0.020 ± 0.005 ^b^	0.017 ± 0.006 ^a^	0.010 ± 0.001 ^a^	0.030 ± 0.006 ^a^	0.035 ± 0.005 ^a^	0.010 ± 0.003 ^b^
YG_Pe	M. Etna (*n* = 3)	0.187 ± 0.031 ^c^	0.087 ± 0.006 ^b^	0.091 ± 0.005 ^a^	0.080 ± 0.030 ^c^	0.087 ± 0.006 ^b^	0.023 ± 0.006 ^b^	0.037 ± 0.007 ^b^	0.023 ± 0.006 ^b^	0.047 ± 0.015 ^a^	0.057 ± 0.014 ^b^	0.005 ± 0.002 ^a^
YG_Pe	Marsala (*n* = 3)	0.043 ± 0.006 ^a^	0.027 ± 0.006 ^a^	0.070 ± 0.010 ^a^	0.010 ± 0.002 ^a^	<LOQ	0.010 ± 0.002 ^a^	<LOQ	<LOQ	<LOQ	<LOQ	<LOQ
PG_J	Stromboli (*n* = 3)	0.027 ± 0.006 ^b^	0.010 ± 0.002 ^a^	0.053 ± 0.006 ^a^	<LOQ	0.006 ± 0.001 ^a^	<LOQ	<LOQ	<LOQ	<LOQ	<LOQ	<LOQ
PG_J	M. Etna (*n* = 3)	0.053 ± 0.006 ^c^	0.043 ± 0.006 ^b^	0.120 ± 0.010 ^b^	<LOQ	0.013 ± 0.006 ^b^	<LOQ	<LOQ	<LOQ	<LOQ	0.006 ± 0.001	<LOQ
PG_J	Marsala (*n* = 3)	0.006 ± 0.001 ^a^	<LOQ	0.013 ± 0.006 ^a^	<LOQ	<LOQ	<LOQ	<LOQ	<LOQ	<LOQ	<LOQ	<LOQ
PG_Pu	Stromboli (*n* = 3)	0.057 ± 0.006 ^b^	0.047 ± 0.006 ^a^	0.153 ± 0.012 ^b^	0.006 ± 0.001 ^a^	0.017 ± 0.006 ^a^	0.017 ± 0.006 ^a^	0.004 ± 0.001 ^a^	0.006 ± 0.001 ^a^	0.023 ± 0.006 ^a^	0.004 ± 0.001 ^a^	0.005 ± 0.001 ^a^
PG_Pu	M. Etna (*n* = 3)	0.073 ± 0.015 ^b^	0.113 ± 0.006 ^b^	0.247 ± 0.025 ^b^	0.013 ± 0.006 ^a^	0.030 ± 0.010 ^b^	0.017 ± 0.006 ^a^	0.005 ± 0.001 ^a^	0.005 ± 0.001 ^a^	0.027 ± 0.006 ^a^	0.013 ± 0.006 ^b^	0.013 ± 0.006 ^b^
PG_Pu	Marsala (*n* = 3)	0.023 ± 0.006 ^a^	<LOQ	0.043 ± 0.006 ^a^	<LOQ	<LOQ	<LOQ	<LOQ	<LOQ	<LOQ	<LOQ	<LOQ
PG_Pe	Stromboli (*n* = 3)	0.083 ± 0.006 ^b^	0.080 ± 0.010 ^b^	0.250 ± 0.036 ^b^	0.027 ± 0.006 ^a^	0.047 ± 0.006 ^a^	0.033 ± 0.006 ^b^	0.013 ± 0.006 ^b^	0.013 ± 0.006 ^a^	0.043 ± 0.006 ^a^	0.023 ± 0.006 ^a^	0.013 ± 0.006 ^a^
PG_Pe	M. Etna (*n* = 3)	0.133 ± 0.015 ^c^	0.167 ± 0.015 ^c^	0.407 ± 0.025 ^c^	0.053 ± 0.006 ^b^	0.077 ± 0.006 ^b^	0.037 ± 0.006 ^b^	0.007 ± 0.002 ^a^	0.013 ± 0.006 ^a^	0.070 ± 0.010 ^b^	0.057 ± 0.012 ^b^	0.027 ± 0.012 ^b^
PG_Pe	Marsala (*n* = 3)	0.043 ± 0.006 ^a^	0.013 ± 0.006 ^a^	0.083 ± 0.006 ^a^	0.013 ± 0.006 ^a^	<LOQ	0.013 ± 0.006 ^a^	<LOQ	<LOQ	<LOQ	<LOQ	<LOQ

L, Lemon; M, Mandarin; YG, Yellow Grapefruit; PG, Pink Grapefruit; J, Juice; Pu, Pulp; Pe, Peel; Superscript letters (a, b, c) indicate statistically significant differences (*p* < 0.05) among samples from Stromboli, Etna, and Marsala.

## Data Availability

Data will be made available on request.

## Supplementary Materials

The following supporting information can be downloaded at https://www.mdpi.com/article/10.3390/foods15173008/s1. Table S1: Analytical performance parameters for the determination of elements in citrus samples: linearity (R^2^), limits of detection (LOD), limits of quantification (LOQ), accuracy, repeatability, and intermediate precision evaluated using SRM 1570a (Spinach Leaves); Table S2: Analytical performance parameters for the determination of elements in soil samples: linearity (R^2^), limits of detection (LOD), limits of quantification (LOQ), accuracy, repeatability, and intermediate precision evaluated using CRM048 (Sandy Soil); Table S3: Element transfer rate (%) in peel, pulp, and juice of citrus fruits samples from volcanic (Stromboli, Etna) and non-volcanic (Marsala) areas. Transfer rates were calculated using the measured concentrations on an as-is basis; Table S4: PCA loading matrix and communalities (h^2^) for variables in the soil dataset; Table S5: PCA loading matrix and communalities (h^2^) for variables in the whole citrus dataset; Table S6: Standardized and unstandardized canonical discriminant function coefficients derived from linear discriminant analysis (LDA); Table S7: Classification results from the original LDA classification and leave-one-out cross-validation (LOOCV); Table S8: Essential and potentially toxic elements in citrus juice (200 mL): percentage contributions to nutrient reference values (NRVs) and toxicological reference values (TRVs) including TDI, TWI, PTWI, RfD, MRL, and BMDL.

## Abbreviations

The following abbreviations are used in this manuscript:

AI	Adequate Intake
Al	Aluminum
As	Arsenic
a.s.l.	Above Sea Level
Ca	Calcium
Cd	Cadmium
Co	Cobalt
Cr	Chromium
Cu	Copper
EDI	Estimated Daily Intake
EFSA	European Food Safety Authority
EU	European Union
FAO	Food and Agriculture Organization
Fe	Iron
Hg	Mercury
ICP-MS	Inductively Coupled Plasma Mass Spectrometry
K	Potassium
KMO	Kaiser–Meyer–Olkin
LDA	Linear Discriminant Analysis
LOD	Limit of Detection
LOOCV	Leave-One-Out Cross-Validation
LOQ	Limit of Quantification
Mg	Magnesium
Mn	Manganese
Mo	Molybdenum
Na	Sodium
NRV	Nutrient Reference Value
P	Phosphorus
PCA	Principal Component Analysis
Pb	Lead
PDO	Protected Designation of Origin
PGI	Protected Geographical Indication
Sb	Antimony
Se	Selenium
TDI	Tolerable Daily Intake
Ti	Titanium
TRV	Toxicological Reference Value
TWI	Tolerable Weekly Intake
V	Vanadium
WHO	World Health Organization
Zn	Zinc
