# Supplementary material for: Multi-Element Analysis of Sicilian Citrus Fruits from Volcanic and Non-Volcanic Areas for Geographical Authentication and Food Safety Assessment
Source: Foods. 2026 Aug 26;15(17):3008. doi: 10.3390/foods15173008 (PMC13565099; doi:10.3390/foods15173008)
Supplement: Supplementary file 1 [file foods-15-03008-s001.zip › foods-4475105-supplementary.pdf]

Supplementary Materials

Multi-Element Analysis of Sicilian Citrus Fruits from Volcanic and Non-Volcanic Areas for Geographical Authentication and Food Safety Assessment

Giuseppa Di Bella [1], Vincenzo Nava [1], Angela Giorgia Potortì [1,*], Roberto Sturniolo [1,2] and Vincenzo Lo Turco [1]

[1] Department of Biomedical and Dental Sciences and of Morphological and Functional Images (BIOMORF), University of Messina, Viale Palatucci 13, 98168 Messina, Italy; gdibella@unime.it (G.D.B.); vnava@unime.it (V.N.); roberto.sturniolo@studenti.unime.it (R.S.); vloturco@unime.it (V.L.T.)

[2] Department of Chemical, Biological, Pharmaceutical, and Environmental Sciences (CHIBIOFARAM), University of Messina, Viale F. Stagno d'Alcontres 31, 98166 Messina, Italy

* Correspondence: agpotorti@unime.it; Tel.: +39-090-6766992

Table S1. Analytical performance parameters for the determination of elements in citrus samples: linearity (R^2), limits of detection (LOD), limits of quantification (LOQ), accuracy, repeatability, and intermediate precision evaluated using SRM 1570a (Spinach Leaves).

Element	R2	LOD (µg·L-1)	LOQ (µg·L-1)	Accuracy (%)	Repeatability (RSD, %)	Intermediate precision (RSD, %)
Al	0.9995	0.003	0.010	97.45	1.1	1.2
As	0.9997	0.001	0.003	98.53	1.3	1.4
Ca	0.9984	0.303	1.000	92.50	1.1	1.3
Cd	0.9998	0.001	0.003	100.34	1.2	1.3
Co	0.9996	0.001	0.003	98.73	1.3	1.5
Cr*	0.9995	0.001	0.003	98.00	0.7	0.8
Cu	0.9997	0.003	0.010	97.05	0.6	0.7
Fe*	0.9997	0.012	0.040	97.00	0.5	0.7
Hg	0.9998	0.001	0.003	96.30	0.6	0.7
K	0.9983	0.515	1.700	95.03	1.2	1.4
Mg	0.9993	0.152	0.502	97.83	1.2	1.3
Mn	0.9997	0.003	0.010	98.82	1.1	1.2
Mo*	0.9994	0.003	0.010	97.50	0.5	0.7
Na	0.9986	0.205	0.677	92.56	1.2	1.3
Ni	0.9995	0.001	0.003	99.21	0.5	0.7
P	0.9990	0.198	0.653	99.15	1.2	1.4
Pb	0.9999	0.001	0.003	105.00	0.7	0.9
Sb*	0.9994	0.001	0.003	98.00	1.0	1.2
Se	0.9994	0.001	0.003	97.48	0.8	1.0
Ti*	0.9995	0.003	0.010	97.50	0.6	0.7
V	0.9994	0.001	0.003	95.78	0.7	0.9
Zn	0.9996	0.010	0.033	96.96	1.1	1.2

Calibration curves were established using six concentration levels of external calibration standards. Linearity was assessed by the coefficient of determination (R^2). Instrumental limits of detection (LOD) and limits of quantification (LOQ) were calculated as 3.3σ/S and 10σ/S, respectively, where σ is the standard deviation of the blank signal and S is the slope of the calibration curve. Method LOQs expressed in matrix units ($\mu g \cdot Kg^{-1}$) can be obtained by applying a conversion factor of 50, derived for the sample mass (0.5 g) and final digestion volume (25 mL). Accuracy, repeatability and intermediate precision were evaluated using certified reference materials. Elements marked with an asterisk were evaluated by spike recovery because certified values were not available in the reference material.

Table S2. Analytical performance parameters for the determination of elements in soil samples: linearity (R^2), limits of detection (LOD), limits of quantification (LOQ), accuracy, repeatability, and intermediate precision evaluated using CRM048 (Sandy Soil).

Element	R2	LOD (µg·L-1)	LOQ (µg·L-1)	Accuracy (%)	Repeatability (RSD, %)	Intermediate precision (RSD, %)
Al	0.9996	0.004	0.013	99.19	1.7	1.9
As	0.9998	0.001	0.003	97.12	1.7	2.1
Ca	0.9986	0.315	1.040	93.17	1.4	1.8
Cd	0.9999	0.001	0.003	100.65	1.2	1.6
Co	0.9995	0.004	0.013	98.17	1.5	1.8
Cr	0.9994	0.003	0.010	97.89	1.1	1.3
Cu	0.9995	0.003	0.010	98.81	0.9	1.3
Fe	0.9998	0.025	0.083	98.99	0.8	0.9
Hg	0.9999	0.001	0.003	102.23	0.7	1.2
K	0.9981	0.677	2.234	92.90	1.8	2.5
Mg	0.9994	0.278	0.917	99.12	1.6	1.9
Mn	0.9995	0.004	0.013	97.08	1.3	1.8
Mo	0.9997	0.002	0.007	97.89	0.9	1.3
Na	0.9984	0.509	1.680	92.72	1.2	2.1
Ni	0.9998	0.001	0.003	98.67	0.6	1.4
P	0.9992	0.277	0.914	97.50	1.4	1.7
Pb	0.9999	0.001	0.003	101.56	0.9	1.3
Sb	0.9993	0.003	0.010	97.40	1.6	1.9
Se	0.9995	0.003	0.010	95.82	0.7	1.1
Ti	0.9994	0.003	0.010	97.97	0.6	1.0
V	0.9995	0.004	0.013	96.13	1.1	1.4
Zn	0.9997	0.013	0.043	96.24	1.4	1.6

Calibration curves were established using six concentration levels of external calibration standards. Linearity was assessed by the coefficient of determination (R^2). Instrumental limits of detection (LOD) and limits of quantification (LOQ) were calculated as $3.3\sigma/S$ and $10\sigma/S$, respectively, where σ is the standard deviation of the blank signal and S is the slope of the calibration curve. Method LOQs expressed in matrix units ($\mu g \cdot Kg^{-1}$) can be obtained by applying a conversion factor of 5000, derived for the sample mass (0.5 g), final digestion volume (25 mL), and the additional 1:100 dilution prior to ICP-MS analysis. Accuracy, repeatability and intermediate precision were evaluated using certified reference materials.

Table S3. Element transfer rate (%) in peel, pulp, and juice of citrus fruits samples from volcanic (Stromboli, Etna) and non-volcanic (Marsala) areas. Transfer rates were calculated using the measured concentrations on an as-is basis.

Samples	Origin	K	P	Ca	Mg	Na	Fe	Zn	Cu	Mn	Al	Mo
L_J	Stromboli	9.1	7.7	1.7	0.6	0.3	<0.05	0.8	0.6	<0.05	<0.05	3.9
L_J	Etna	11.1	6.8	1.3	0.3	0.1	<0.05	0.7	0.2	<0.05	<0.05	3.2
L_J	Marsala	22.1	12.2	1.9	0.4	0.1	<0.05	0.9	0.7	<0.05	<0.05	1.9
L_Pu	Stromboli	10.7	11.0	3.7	1.3	0.6	<0.05	4.2	1.1	<0.05	<0.05	6.8
L_Pu	Etna	14.1	9.6	2.9	0.7	0.2	<0.05	2.4	0.5	<0.05	<0.05	6.3
L_Pu	Marsala	28.0	21.0	4.1	0.9	0.2	<0.05	4.6	1.2	<0.05	<0.05	3.8
L_Pe	Stromboli	12.4	14.2	5.6	1.7	1.0	<0.05	4.9	1.3	<0.05	<0.05	9.4
L_Pe	Etna	15.5	12.1	4.1	0.9	0.2	<0.05	3.3	0.7	<0.05	<0.05	10.0
L_Pe	Marsala	29.9	27.2	5.9	1.2	0.4	<0.05	6.1	1.5	<0.05	<0.05	9.0
M_J	Stromboli	8.2	8.9	3.3	0.7	0.4	<0.05	0.8	0.7	<0.05	<0.05	7.7
M_J	Etna	9.7	8.6	2.8	0.5	0.2	<0.05	0.6	0.6	<0.05	<0.05	5.0
M_J	Marsala	21.9	15.7	3.5	0.6	0.2	<0.05	0.6	0.7	<0.05	<0.05	6.3
M_Pu	Stromboli	9.1	12.4	4.2	1.5	0.7	<0.05	3.3	1.2	0.1	<0.05	10.3
M_Pu	Etna	10.6	10.8	3.7	0.7	0.2	<0.05	2.3	0.8	<0.05	<0.05	6.8
M_Pu	Marsala	24.9	21.8	4.6	1.1	0.3	<0.05	4.2	1.6	0.1	<0.05	10.8
M_Pe	Stromboli	9.7	14.0	5.5	1.5	0.6	<0.05	4.2	1.6	0.1	<0.05	14.2
M_Pe	Etna	11.9	13.3	4.8	0.9	0.3	<0.05	2.8	1.3	<0.05	<0.05	8.1
M_Pe	Marsala	27.2	24.2	5.9	1.1	0.3	<0.05	5.7	2.0	0.1	<0.05	14.6
YG_J	Stromboli	7.7	11.1	3.0	1.7	0.3	<0.05	2.9	0.8	<0.05	<0.05	12.0
YG_J	Etna	10.3	10.4	2.4	0.6	0.2	<0.05	2.8	0.5	<0.05	<0.05	4.5
YG_J	Marsala	16.2	13.5	3.3	1.2	0.1	<0.05	3.6	1.0	<0.05	<0.05	1.9
YG_Pu	Stromboli	9.0	13.3	4.7	1.9	0.5	<0.05	3.3	1.0	<0.05	<0.05	21.5
YG_Pu	Etna	11.2	12.0	3.3	0.8	0.3	<0.05	3.4	0.8	<0.05	<0.05	8.1
YG_Pu	Marsala	20.4	19.3	5.0	1.4	0.2	<0.05	4.5	1.3	<0.05	<0.05	6.3
YG_Pe	Stromboli	9.4	13.6	7.0	2.2	0.6	<0.05	3.7	1.3	0.1	<0.05	28.8
YG_Pe	Etna	13.1	12.7	5.5	1.1	0.4	<0.05	4.8	1.2	0.1	<0.05	14.0
YG_Pe	Marsala	21.8	21.4	7.3	1.6	0.2	<0.05	4.7	1.6	0.1	<0.05	12.1
PG_J	Stromboli	7.0	10.4	2.8	1.5	0.3	<0.05	2.6	1.0	<0.05	<0.05	6.9
PG_J	Etna	10.6	10.5	3.3	1.3	0.2	<0.05	2.1	0.7	<0.05	<0.05	8.1
PG_J	Marsala	15.9	11.6	1.7	0.4	0.1	<0.05	3.2	1.4	<0.05	<0.05	2.5
PG_Pu	Stromboli	8.1	12.6	4.2	1.6	0.4	<0.05	3.1	1.2	<0.05	<0.05	13.3
PG_Pu	Etna	11.8	13.4	4.9	1.4	0.3	<0.05	2.5	0.9	<0.05	<0.05	14.0
PG_Pu	Marsala	16.4	18.0	2.4	0.5	0.1	<0.05	4.2	1.7	<0.05	<0.05	4.4
PG_Pe	Stromboli	8.1	12.6	4.2	1.6	0.4	<0.05	3.1	1.2	<0.05	<0.05	13.3
PG_Pe	Etna	11.8	13.4	4.9	1.4	0.3	<0.05	2.5	0.9	<0.05	<0.05	14.0
PG_Pe	Marsala	16.4	18.0	2.4	0.5	0.1	<0.05	4.2	1.7	<0.05	<0.05	4.4

L, Lemon; M, Mandarin; YG, Yellow Grapefruit; PG, Pink Grapefruit; J, Juice; Pu, Pulp; Pe, Peel; -, there is no transfer.

Table S3. Continued

Samples	Origin	Se	Ni	Cr	V	Co	Pb	As	Sb	Ti	Cd	Hg
L_J	Stromboli	3.16	0.06	0.31	-	-	-	-	-	-	-	-
L_J	Etna	0.28	0.06	0.08	-	-	-	-	-	-	-	-
L_J	Marsala	1.91	0.01	-	-	-	-	-	-	-	-	-
L_Pu	Stromboli	11.05	0.32	2.62	<0.05	0.1	0.1	-	-	0.5	0.8	-
L_Pu	Etna	1.31	0.12	0.55	<0.05	0.1	<0.05	0.2	-	1.0	0.8	-
L_Pu	Marsala	7.27	0.08	0.09	-	-	-	-	-	-	-	-
L_Pe	Stromboli	21.05	0.52	3.83	0.1	0.2	0.3	0.3	0.9	1.0	1.4	25.8
L_Pe	Etna	2.05	0.25	0.89	0.0	0.2	0.1	0.4	1.1	2.1	2.4	24.8
L_Pe	Marsala	17.27	0.14	0.13	-	-	-	-	-	-	-	-
M_J	Stromboli	3.51	0.03	0.37	-	-	-	-	-	-	-	-
M_J	Etna	0.45	0.02	0.06	-	-	-	-	-	-	-	-
M_J	Marsala	-	-	-	-	-	-	-	-	-	-	-
M_Pu	Stromboli	9.8	0.1	1.2	<0.05	-	-	-	-	0.5	-	11.9
M_Pu	Etna	1.2	0.1	0.5	<0.05	-	-	0.1	-	1.4	-	12.2
M_Pu	Marsala	7.3	<0.05	<0.05	-	-	-	-	-	-	-	-
M_Pe	Stromboli	14.0	0.4	2.1	0.1	0.1	0.1	0.3	0.5	1.2	0.9	37.9
M_Pe	Etna	1.7	0.3	0.8	0.1	0.2	<0.05	0.3	0.4	2.5	0.6	34.8
M_Pe	Marsala	14.5	0.1	0.1	<0.05	-	-	-	-	-	-	-
YG_J	Stromboli	9.8	0.1	1.1	-	0.1	-	-	-	-	-	-
YG_J	Etna	2.1	<0.05	0.1	-	0.1	-	-	-	-	-	-
YG_J	Marsala	3.6	<0.05	<0.05	-	-	-	-	-	-	-	-
YG_Pu	Stromboli	16.1	0.4	4.4	<0.05	0.3	0.1	0.1	-	0.5	1.3	15.4
YG_Pu	Etna	3.3	0.2	0.4	<0.05	0.2	<0.05	0.1	-	1.4	0.7	7.5
YG_Pu	Marsala	10.9	<0.05	0.1	-	-	-	-	-	-	-	-
YG_Pe	Stromboli	18.9	0.6	5.7	0.1	0.4	0.2	0.4	0.5	1.6	4.2	21.1
YG_Pe	Etna	4.2	0.2	0.7	0.1	0.5	0.1	0.6	1.0	3.9	2.9	11.6
YG_Pe	Marsala	15.8	0.1	0.1	<0.05	-	<0.05	-	-	-	-	-
PG_J	Stromboli	5.6	0.1	2.0	-	<0.05	-	-	-	-		-
PG_J	Etna	1.2	0.1	0.9	-	0.1	-	-	-	-	0.3	-
PG_J	Marsala	2.2	-	<0.05	-	-	-	-	-	-	-	-
PG_Pu	Stromboli	11.9	0.3	5.7	<0.05	0.1	0.1	0.1	0.3	1.2	0.5	11.2
PG_Pu	Etna	1.7	0.3	1.9	<0.05	0.2	<0.05	0.1	0.2	2.2	0.7	23.2
PG_Pu	Marsala	8.5	-	0.1	-	-	-	-	-	-	-	-
PG_Pe	Stromboli	17.5	0.5	9.3	<0.05	0.3	0.3	0.3	0.7	2.3	2.7	28.1
PG_Pe	Etna	3.0	0.5	3.1	0.1	0.4	0.1	0.1	0.6	5.8	2.8	46.4
PG_Pe	Marsala	15.8	<0.05	0.2	<0.05	-	<0.05	-	-	-	-	-

L, Lemon; M, Mandarin; YG, Yellow Grapefruit; PG, Pink Grapefruit; J, Juice; Pu, Pulp; Pe, Peel; -, there is no transfer.

Table S4. PCA loading matrix and communalities (h2) for variables in the soil dataset.

	**PC1**	**PC2**	**h^2**
Al	**0.999**	-0.007	0.992
K	**0.996**	0.029	0.991
Fe	**0.986**	-0.110	0.942
Na	**-0.959**	0.264	0.989
Mg	**-0.953**	0.183	0.985
Cr	**-0.953**	0.285	0.943
As	**-0.923**	0.178	0.966
P	**0.915**	0.393	0.998
Pb	**-0.909**	0.397	0.990
Cu	0.654	**0.718**	0.984
Mn	0.693	**0.697**	0.883

Bold values indicate the dominant variables in each factor.

Table S5. PCA loading matrix and communalities (h2) for variables in the whole citrus dataset.

	PC1	PC2	PC3	PC4	PC5	h^2
Co	0.989	-0.021	0.044	-0.022	-0.022	0.982
Ti	0.980	-0.039	-0.002	0.097	0.019	0.972
Cd	0.978	-0.037	-0.011	-0.092	0.049	0.969
Sb	0.959	-0.054	-0.206	-0.014	0.055	0.968
P	0.951	0.125	0.061	0.192	-0.107	0.973
As	0.927	-0.157	-0.220	0.103	-0.016	0.973
Hg	0.925	-0.003	-0.112	0.147	0.165	0.917
Se	0.920	-0.019	0.137	-0.074	-0.290	0.955
Mo	0.888	-0.028	0.261	0.218	0.103	0.915
V	0.847	-0.393	0.067	0.197	0.125	0.931
Ni	0.820	0.464	-0.097	0.056	-0.064	0.904
Al	0.812	0.387	0.042	-0.343	-0.184	0.963
Pb	0.811	-0.260	0.037	-0.254	0.319	0.892
Cu	0.754	-0.350	0.261	0.127	-0.247	0.836
Cr	0.713	0.140	0.325	-0.505	0.117	0.904
Zn	0.685	-0.037	0.281	-0.424	-0.365	0.863
K	0.593	0.519	-0.563	0.151	0.001	0.961
Mn	0.576	-0.104	0.363	0.531	0.378	0.900
Na	0.225	0.668	-0.216	0.441	-0.363	0.869
Fe	0.235	0.658	-0.404	-0.307	0.463	0.959
Mg	-0.065	0.618	0.745	-0.060	0.169	0.974
Ca	-0.454	0.563	0.633	0.241	0.005	0.983

Bold values indicate the dominant variables in each factor.

Table S6. Standardized and unstandardized canonical discriminant function coefficients derived from linear discriminant analysis (LDA).

	**Standardized coefficients**		**Unstandardized coefficients**	
	**F1**	**F2**	**F1**	**F2**
K	+5.035	+8.624	+34.991	+59.936
P	+1.996	-3.789	+22.183	-42.107
Ca	+3.376	+7.826	+16.877	+39.125
Mg	-4.492	-6.255	-13.941	-19.413
Na	-3.464	-8.826	-12.172	-31.010
Fe	+0.990	-0.484	+2.450	-1.196
Zn	+1.138	+1.817	+4.939	+7.887
Cu	+0.387	+6.817	+1.942	+34.222
Mn	+0.402	-1.057	+1.366	-3.594
Al	+4.603	-0.800	+10.946	-1.902
Mo	+0.689	+0.394	+1.967	+1.124
Se	+0.409	+3.402	+1.275	+10.593
Ni	-3.913	+1.375	-7.307	+2.567
Cr	+0.353	-2.683	+0.604	-4.588
V	+3.330	-1.323	+5.703	-2.267
Co	+2.018	+0.864	+3.368	+1.442
Pb	-1.150	-1.083	-1.478	-1.391
As	+1.848	-1.223	+4.983	-3.296
Sb	-1.673	-1.470	-4.282	-3.763
Ti	-0.770	-2.282	-2.542	-7.530
Cd	-3.578	+3.099	-5.896	+5.106
Hg	+2.904	+2.682	+9.311	+8.600
Costant			-322.918	-234.550

Table S7. Classification results from the original LDA classification and leave-one-out cross-validation (LOOCV).

			Predicted Group Membership			
		**Geographic Origin**	**Stromboli**	**Etna**	**Marsala**	**Total**
Original	Count	Stromboli	13	0	0	13
		Etna	0	13	0	13
		Marsala	0	0	13	13
	%	Stromboli	100	0	0	100
		Etna	0	100	0	100
		Marsala	0	0	100	100
Cross-validated	Count	Stromboli	13	0	0	13
		Etna	0	13	0	13
		Marsala	0	0	13	13
	%	Stromboli	100	0	0	100
		Etna	0	100	0	100
		Marsala	0	0	100	100

Table S8. Essential and potentially toxic elements in citrus juice (200 mL): percentage contributions to nutrient reference values (NRVs) and toxicological reference values (TRVs) including TDI, TWI, PTWI, RfD, MRL, and BMDL.

Element	NRVs/TRVs	References	Lemon Juice			Mandarin Juice			Yellow Grapefruit Juice			Pink Grapefruit Juice		
			Stromboli	Etna	Marsala	Stromboli	Etna	Marsala	Stromboli	Etna	Marsala	Stromboli	Etna	Marsala
K	NRV: 2000 mg/day	Reg. UE n 1169/2011	17.28	15.56	13.80	15.69	13.59	13.67	14.68	14.35	10.09	13.26	14.79	9.92
P	NRV: 700 mg/day	Reg. UE n 1169/2011	4.91	4.86	3.08	5.69	6.12	3.95	7.11	7.42	3.41	6.63	7.52	2.92
Ca	NRV: 800 mg/day	Reg. UE n 1169/2011	2.93	2.17	4.08	5.77	4.73	7.54	5.14	4.09	7.15	4.82	5.63	3.64
Mg	NRV: 375 mg/day	Reg. UE n 1169/2011	3.66	2.46	4.11	4.29	3.79	5.49	10.40	4.29	11.53	9.26	10.06	3.63
Na	AI: 2000 mg/day	EFSA. 2019	0.09	0.05	0.07	0.14	0.10	0.10	0.11	0.10	0.05	0.09	0.10	0.05
Fe	NRV: 14 mg/day	Reg. UE n 1169/2011	3.26	2.74	2.13	2.93	1.59	2.13	4.37	1.03	2.37	3.91	4.34	2.13
Zn	NRV: 10 mg/day	Reg. UE n 1169/2011	1.32	1.62	1.00	1.32	1.46	0.66	4.86	6.46	3.78	4.30	4.82	3.38
Cu	NRV: 1 mg/day	Reg. UE n 1169/2011	6.60	4.80	4.60	8.60	12.40	4.40	9.20	10.80	6.00	11.60	15.20	8.60
Mn	NRV: 2 mg/day	Reg. UE n 1169/2011	2.20	2.60	1.00	5.20	2.20	3.20	3.10	3.20	1.90	1.90	2.70	0.30
Mo	NRV: 50 µg/day	Reg. UE n 1169/2011	12.00	19.20	4.00	24.00	29.20	13.20	37.20	26.80	4.00	21.20	48.00	5.20
Se	NRV: 55 µg/day	Reg. UE n 1169/2011	5.45	4.73	1.82	6.18	7.27	1.82	17.09	33.82	3.64	9.82	19.27	2.18
Cr	NRV: 40 µg/day	Reg. UE n 1169/2011	4.00	5.50		5.00	4.00	2.50	15.00	8.50	5.00	26.50	60.00	6.50
Ni	TDI: 13 µg/kg b.w./day	EFSA, 2020	0.22	0.51	0.09	0.11	0.18		0.37	0.29		0.22	0.95	
Al	TWI: 1 mg/kg b.w./week	EFSA, 2008	0.12	0.15	0.08	0.11	0.13	0.05	0.22	0.25	0.09	0.33	0.49	0.07
V	RfD: 7 µg/kg b.w./day	EPA, 2006												
Co	MRL: 20 µg/kg b.w./day	ATSDR, 2024							0.14	0.14		0.09	0.19	0.01
Pb	BMDL01: 0.5 µg/kg b.w./day	EFSA, 2010												
As	BMDL01: 0.3 µg/kg b.w./day	EFSA, 2009												
Sb	TDI: 6 µg/kg b.w./day	WHO, 2003												
Ti	-	-												
Cd	TWI: 2.5 µg/kg b.w./week	EFSA, 2012a											4.00	
Hg	PTWI: 4 µg/kg b.w./week	EFSA, 2012b												

TDI (Tolerable Daily Intake), TWI (Tolerable Weekly Intake), PTWI (Provisional Tolerable Weekly Intake), RfD (Reference Dose), MRL (Minimal Risk Level), BMDL (Benchmark Dose Lower Confidence Limit).
